# Selective synthesis of *α*- and *β*-glycosides of *N*-acetyl galactosamine using rare earth metal triflates

**DOI:** 10.3389/fchem.2022.1029911

**Published:** 2022-10-26

**Authors:** Jiajia Wang, Wei Zhang, Wei Cao, Kang Liu, Shihao Su, Jing Ma, Xia Li

**Affiliations:** ^1^ Joint National Laboratory for Antibody Drug Engineering, The First Affiliated Hospital of Henan University, Henan University, Kaifeng, Henan, China; ^2^ School of Pharmacy, Academy for Advanced Interdisciplinary Studies, Institute of Chemical Biology, Henan University, Kaifeng, Henan, China

**Keywords:** glycosylation, GalNAc, asialoglycoprotein receptor (ASGPR), hydrogen sulfide, selective

## Abstract

Structures containing galactose and GalNAc residues are specifically recognized by asialoglycoprotein receptors, allowing them to selectively internalize by hepatocytes for drug-targeting delivery. However, methods for direct synthesis of GalNAc glycosides are still challenging due to the poor participating group of 2-acetamido. Here, we develop a facile strategy to synthesize various GalNAc glycosides by employing a series of rare earth metal triflates, and the results demonstrate that both *α*-glycosides and *β*-glycosides of GalNAc can be obtained by conducting with Hf(OTf)_4_ and Sc(OTf)_3_, respectively. These applicable results indicate that any interested GalNAc-containing substrates could be prepared by this simple strategy.

## Introduction

Recognition between carbohydrates and proteins has attracted great attention because of their roles in fundamental biological processes, such as cell signaling, fertilization, immune function, and cancer metastasis (Ghazarian et al., 2011). The asialoglycoprotein receptor (ASGP-R) is specifically expressed on the membrane of mammalian hepatocytes, which selectively recognizes terminal galactose and *N*-acetyl-galactosamine (GalNAc) residues on glycoconjugates, allowing for targeting the delivery of effecting drugs and genes to the liver (Baenziger and Maynard, 1980; Delannoy et al., 1996; Lee et al., 2012; Zhang et al., 2015). A large amount of galactose/GalNAc-terminated ligands have been widely exploited to selectively recognize and detect hepatocytes. In addition, it has been confirmed that a strategy of multivalent events known as the “cluster glycoside effect” could effectively increase the sugar–protein interactions (Pujol et al., 2012; Shang et al., 2018; Ahn et al., 2021). Hence, the construction of various isomers of GalNAc glycosides is useful for chemical synthesis and biologically functional investigation. For example, GalNAc-*α*-Bn is initially used as an inhibitor of mucin-type *O*-glycosylation in cellular experiments with high concentrations of 2–5 mM, as well as an acceptor substrate for the synthesis of Tn *in vitro* (Delannoy et al., 1996). Interestingly, it is discovered that a low concentration of Ac_3_GalNAc-α-Bn could be exploited as cellular *O*-glycome reporter/amplification (CORA) to profile and amplify mucin-type *O*-glycans in living cells (Kudelka et al., 2016). Based on this success, a large variety of derivatives containing fluorescent and bio-orthogonal groups at site 1 were successfully synthesized and evaluated. Moreover, well-synthesized 3-azido-propyl 2-acetamido-2-deoxy-*α*-galactoside (GalNAc-*α*-ProN_3_), as the acceptor, is used to prepare mono- and di-fluorinated Thomsen–Friedenreich (T) antigens *via* the chemoenzymatic method (Yan et al., 2013). Alternatively, a large number of *β*-galactose or GalNAc-containing glycoconjugates are developed to target the delivery of a therapeutic agent or a fluorescent probe for imaging due to the high-specific glycosidase enzymes of *β*-galactosidase (Dong et al., 2015; Calatrava-Perez et al., 2016; Szekely et al., 2018). Therefore, it is very essential to develop practical methods to construct *α*- or *β*-linked galactose and GalNAc substrates for biological evaluation *in vitro* and *in vivo*, the configuration of which depends on the need.

Methodologies for the synthesis of galactose-containing glycosides have been well explored by utilizing the effect of neighboring group participation similar to other pyranoses. However, the usage of GalNAc tetraacetate glycoside donors for the preparation of *β*-glycosides of GalNAc is limited because the acetamido group of GalNAc at site 2 easily generates a stable intermediate of oxazoline, preventing direct chemical synthesis. Therefore, for *β*-HexNAc glycosides, temporary protecting groups such as Troc-, phthalimido-, azido, and chloroacetamido, have been well investigated for the preparation of *O*-linked desired products after removing the protecting group and full acetylation. Nevertheless, the direct synthesis of the *β*-linked resultant is still facile and attractive. The first success of direct synthesis involves per-acetylated *N*-acetyl glucopyranosyl chloride and alcohols with heavy metal salts as a promoter, which strongly promoted the development of glycosylation. In addition, oxazoline is another alternative potential donor activated with Lewis acids (Cai et al., 2005), cupric salts (Wittmann and Lennartz, 2002), and rare earth metal triflates (Crasto and Jones, 2004; Krag et al., 2010; Stevenin et al., 2012; Kumari et al., 2016; Frem et al., 2017; Forman et al., 2019; Richards et al., 2020) for chemical glycosylation, but restrictions of preparation and low stability limit its wide usage. To address this issue, strong Lewis acids or Bronsted acids are required for glycosylation, and elevating temperature and microwave are other improvement strategies used to convert oxazoline derivatives to the corresponding glycosides. Of note, among common rare earth metal triflates, hafnium (IV) tetratriflate was found to be a good promoter, which was widely applicable to a variety of reactions, such as glycosylation reactions (Manabe and Ito, 2013; Chen and Zhang, 2017; Wang et al., 2018) and *β*-carbamate ketones (Chen et al., 2022).

Although tremendous success has been achieved in the preparation of *β*-glycosides of *N*-acetyl-D-glucosamine (GlcNAc) with mild and efficient protocols (Christensen et al., 2008; Stevenin et al., 2012; Forman et al., 2019), methodologies for the direct formation of GalNAc conjugates and their biological evaluation are rarely reported. Here, we disclose a facile and simple strategy for the synthesis of *β*-glycosides of GalNAc by detecting a series of rare earth metal triflate promoters and various acceptors. The results demonstrate that Sc(OTf)_3_ is a potential activator providing satisfied conversion with a high *β* ratio, while Hf(OTf)_4_ is used preferentially for selective generation of *α*-configuration products. In addition, a selective and targeted turn-on fluorescent probe for detecting hydrogen sulfide is synthesized and evaluated in three kinds of mammalian cells. We find that it could specifically image H_2_S in HepG2 cells, which further exhibits the potential selectivity of *β*-glycosides of GalNAc.

## Experiment

### General methods

All reagents, except otherwise stated, were used as purchased without further purification. Products were purified by column chromatography packed with silica gel (200–300 mesh). TLC plates (thin layer chromatography, HSGF254) were visualized by using an anisaldehyde chromogenic agent solution (135 ml EtOH + 5 ml H_2_SO_4_ + 1.5 ml AcOH + 3.7 ml anisaldehyde) and heated until colored spots appeared to see if the reaction was complete. ^1^H and ^13^C NMR experiments were recorded on a Bruker 300 or 400 M NMR (nuclear magnetic resonance) instrument to determine the structure and configuration of compounds.

### General procedure for the synthesis of 5-chloro-1-pentanol-GalNAc (2) with various catalysts

Donor **1** (500 mg, 1.28 mmol) and the catalyst (0.64 mmol) were dissolved in dry 1,2-C_2_H_4_Cl_2_ (20 ml), and 5-chloro-1-pentanol (**3**) (0.40 ml, 3.85 mmol) was added. The mixture was refluxed under a N_2_ atmosphere for the indicated time, which was quenched by adding Et_3_N until no further conversion was detected by TLC analysis. The reaction mixture was worked up by diluting it with CH_2_Cl_2_ and washing the organic layer with saturated NaHCO_3_, followed by extraction with CH_2_Cl_2_. The mixture was then dried over anhydrous MgSO_4_ and purified by column chromatography. TLC: R_f_ = 0.55 (hexanes/acetone 1:1).

### Cell culture

For cell culturing, 4T1, PC3, and HepG2 cells were cultured in DMEM (Sigma) supplemented with 10% fetal bovine serum (PAN-Seratech), 100 U/ml penicillin, and 100 mg/ml streptomycin 7860. All cell lines were maintained in a humidified cell incubator at 37°C with an atmosphere of 5% CO_2_.

### Detection of endogenous H_2_S *in vitro* with GalNAc-naphthalimide-azide

4T1, PC3, and HepG2 cells were seeded at a density of 500 cells into glass-bottom cell culture dishes and then treated with 10 μM GNA for 1 h under DMEM conditions. Then, the dishes were washed three times with PBS, and the labeling capability was detected by confocal laser scanning microscopy (Leica TCS SP8 + STED) images.

## Result and discussion

### Various Lewis acid catalysts

**SCHEME 1:**
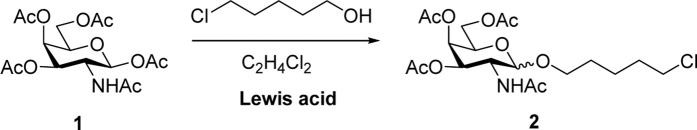
Synthesis of GalNAc *β*-glycosides.

Early in this study, the Jensen Lab discovered that all Lewis acids could promote glycosylation, but Sc(OTf)_3_ and Cu(OTf)_2_ were used as more active catalysts, resulting in a faster and more complete reaction to get benzyl *β*-GlcNAc glycoside (Christensen et al., 2008). Based on these discoveries, we attempt to set up a facile and reliable strategy for constructing *β*-glycosides of GalNAc. Glycosylation was conducted with per-acetylated GalNAc and 5-chloro-1-pentanol as reaction models by using various rare earth metal triflate catalysts. At first, we selected Cu(OTf)_2_ to catalyze the reaction in the solvent of 1,2-C_2_H_4_Cl_2_ at 50°C, which was detected every 4 h (Scheme 1). To our surprise, there were no significant new products observed after 29 h (Entry 1, Table 1). We next tried to elevate the reaction temperature to 90°C for reflux; then, 6 h later, we surprisingly found that all the starting material of Ac_4_GalNAc was completely consumed. Following TLC analysis and NMR spectral characteristics, we discovered that the product was a mixture (*α*:*β* = 40:60), and the point with higher polarity on the TLC plate was confirmed to be α-configuration by NMR analysis (Entry 2, Table 1). Subsequently, all the reactions were conducted at the conditions of 90°C and detected by TLC until there were no more conversions from the starting materials. Of note, if a reaction lasted more than a day and raw material still remained, we regarded the catalyst inefficient.

**TABLE 1:** Results of glycosylation according to Scheme 1 conducted with various Lewis acid catalysts.

Entry	T/°C^a^	Solvent	Cat (50 mol%)	Percentage (*α*:*β*)	Time (h)	Conversion^b^
1	50	1,2-C_2_H_4_Cl_2_	Cu(OTf)_2_	10/10	29	20
2	90	1,2-C_2_H_4_Cl_2_	Cu(OTf)_2_	40/60	6	100
3	90	1,2-C_2_H_4_Cl_2_	Al(OTf)_3_	40/60	12	100
4	90	1,2-C_2_H_4_Cl_2_	In(OTf)_3_	40/60	15	100
5	90	1,2-C_2_H_4_Cl_2_	Hf(OTf)_4_	90/10	12	100
6	90	1,2-C_2_H_4_Cl_2_	Zn(OTf)_2_	50/50	12	100
7	90	1,2-C_2_H_4_Cl_2_	Gd(OTf)_3_	40/60	24	100
8	90	1,2-C_2_H_4_Cl_2_	AgOTf	50/50	12	100
9	90	1,2-C_2_H_4_Cl_2_	Er(OTf)_3_	40/60	12	100
10	90	1,2-C_2_H_4_Cl_2_	Sc(OTf)_3_	10/90	12	100
11	90	1,2-C_2_H_4_Cl_2_	Ce(OTf)_3_	40/60	24	100
12	90	1,2-C_2_H_4_Cl_2_	Fe(OTf)_3_	50/50	12	100
13	90	1,2-C_2_H_4_Cl_2_	Yb(OTf)_3_	30/70	6	100
14	90	1,2-C_2_H_4_Cl_2_	NaOTf	20/60	24	80
15	90	1,2-C_2_H_4_Cl_2_	Y(OTf)_3_	40/50	25	90
16	90	1,2-C_2_H_4_Cl_2_	La(OTf)_3_	20/60	24	80
17	90	1,2-C_2_H_4_Cl_2_	Sa (OTf)_3_	30/40	30	70

^a^ Oil bath temperature.

^b^ Shown by TLC.

After carefully analyzing the reactions catalyzed by various rare earth metal triflates, we discovered that most of these catalysts could completely convert the donor, such as Cu(OTf)_2_, Al(OTf)_3_, In(OTf)_3_, Hf(OTf)_4_, Zn(OTf)_2_, Gd(OTf)_3_, AgOTf, Er(OTf)_3_, Sc(OTf)_3_, Ce(OTf)_3_, Fe(OTf)_3_, and Yb(OTf)_3_ (entries 2–13, Table 1). Also, there were some catalysts that were difficult to complete the reaction over a longer period of time, including NaOTf, Y(OTf)_3_, La(OTf)_3_, and Sa (OTf)_3_ (entries 14–17, Table 1). Based on the aforementioned results, we concluded that the ratio of *α*/*β* configurations varies from one catalyst to another, and Sc(OTf)_3_ is the best catalyst to promote a *β*-configurational product with an *α*/*β* ratio of 10/90 (entry 10, Table 1). Moreover, we surprisingly found that Hf(OTf)_4_ could provide a satisfying *α*-product with an *α*/*β* ratio of 90/10 after refluxing for 12 h. The possible mechanisms for these results may be concluded that the *β*-selectivity of Sc(OTf)_3_ probably depends on the kinetic stability, while the products conducted with Hf(OTf)_4_, to some extent, are thermodynamically dependent. To further determine this hypothesis, the reversibility tests were conducted with purified *α*- or *β*-5-chloropentanol-Ac_4_GalNAc or -Ac_4_GlcNAc in the presence of Sc(OTf)_3_ or Hf(OTf)_4_ by refluxing for 24 h. The results demonstrated that both *β*-5-chloropentanol-Ac_4_GalNAc and -Ac_4_GlcNAc could be converted to an *α/β* mixture, suggesting that Hf(OTf)_4_ is easy to form α-products largely based on the thermodynamic catalysis. However, no matter *α/β* GalNAc- or *β* GlcNAc-glycosides, Sc(OTf)_3_ could not reverse the structures of reactants (Supplementary Figure S2). These discoveries further confirm the selectivities of Sc(OTf)_3_ and Hf(OTf)_4_ in the generation of desired *α*- or *β*-configurational products.

### Optimizing reaction conditions

*β*-glycosides of GalNAc or galactose are common structures constructed for different applications, such as substrates of *β*-galactosidase for selective release (Thomas et al., 2011; Abboud et al., 2021). Therefore, we next focus our attention on the optimization of *β*-GalNAc glycosides with various reaction conditions, including changed ratios of the donor and acceptor and the equivalent of Sc(OTf)_3._ Generally, it is well known that the ratios of glycosyl acceptors are important to the conversion and configuration of products. Therefore, various ratios of the donor and acceptor, from 2:1 to 1:10, were employed to determine the conversions and *α/β* ratios of each reaction, all of which were conducted at the conditions of 50 mol% Cat, 90°C, and 1,2-C_2_H_4_Cl_2_ as the solvent. When the ratio of the donor to acceptor was 2:1, the conversion rate was 60% (*α*:*β* = 50: 50) (entry 1, Table 2). However, the reaction conversion increased gradually with the increasing equivalent of 5-chloro-1-pentanol, and the product had a predominant *β*-configuration (entries 1–4, Table 2). In particular, when the ratio of the donor to acceptor was 1:10, the reaction was complete, and the *β*-configurational product accounted for 90% (entry 4, Table 2). Taken together, we conclude that a higher ratio of the acceptor to donor is beneficial for the conversion and generation of *β*-glycosides of GalNAc.

**TABLE 2:** Results of glycosylation conducted with different amounts of the acceptor (5-Cl-1-pentanol).

Entry	T/°C^a^	Solvent	Cat (50 mol%)	D^b^/A^c^	Time	Conversion %^d^ (*α*:*β)*
1	90	1,2-C_2_H_4_Cl_2_	Sc(OTf)_3_	2:1	12	60 (50:50)
2	90	1,2-C_2_H_4_Cl_2_	Sc(OTf)_3_	1:1	12	80 (20:60)
3	90	1,2-C_2_H_4_Cl_2_	Sc(OTf)_3_	1:3	12	90 (20:70)
4	90	1,2-C_2_H_4_Cl_2_	Sc(OTf)_3_	1:10	12	100 (10:90)

^a^ Oil bath temperature.

^b^ Donor: Ac_4_GalNAc.

^c^ Acceptor: 5-Cl-1-pentanol.

^d^ Observed by a TLC plate.

Next, we further investigated the influence of the amount of catalyst on the reaction. All the reactions were conducted at 90 °C for 12 h and with 1,2-C_2_H_4_Cl_2_ as the solvent. When the ratio of the donor to catalyst was 10:1, the conversion rate was only 50% (*α*:*β* = 10:40) (entry 1, Table 3). However, with the increasing amount of the catalyst, we found that the reaction yield increased gradually, as well as the proportion of the *β-*configurational product (entries 1–4, Table 3). However, when the catalytic equivalent was half or equal to that of the donor, there was no obvious influence on the conversion and configuration (entries 4–5, Table 3). All the results disclose that 50 mol% of the catalyst Sc(OTf)_3_ will provide a satisfied conversion with *β*-selective products.

**TABLE 3:** Results of glycosylation conducted with different amounts of the catalyst (Sc(OTf)_3_).

Entry	T/°C^a^	Solvent	Cat	mol (D^b^)/mol (C^c^)	Time	Yield %^d^ (*α*:*β*)
1	90	1,2-C_2_H_4_Cl_2_	Sc(OTf)_3_	10:1	12	50 (10:40)
2	90	1,2-C_2_H_4_Cl_2_	Sc(OTf)_3_	5:1	12	80 (20:60)
3	90	1,2-C_2_H_4_Cl_2_	Sc(OTf)_3_	3:1	12	95 (20:75)
4	90	1,2-C_2_H_4_Cl_2_	Sc(OTf)_3_	2:1	12	100 (10:90)
5	90	1,2-C_2_H_4_Cl_2_	Sc(OTf)_3_	1:1	12	100 (10:90)

^a^ Oil bath temperature.

^b^ Donor: Ac_4_GalNAc.

^c^ Catalyst: Sc(OTf)_3_.

^d^ Observed by a TLC plate.

### Glycosylation with various donors and acceptors

After optimizing the reaction conditions, we next attempted to evaluate this method for a wide application by using different glycosyl donors (Ac_4_2deoGlucose **4**, Ac_4_GlcNAc **5**, 1,3,4,6-tetraacetyl *N*-thioglycolyl-glucosamine **6**, Ac_3_4FGlcNAc **7,** and Ac_4_GalNAc **1**; Table 4) and alcohol acceptors (5-chloro-1-pentanol **3**, 3-bromo-1-propanol **8**, and 4-hydroxyphenylboronic acid pinacol ester **9**). All the reactions were undertaken based on the aforementioned optimized method, specifically, using Sc(OTf)_3_ (50 mol%) as the catalyst and refluxed in 1,2-C_2_H_4_Cl_2_. Based on the results, it was found that HexNAc analogs **4**, **6**, and **7** could provide high *β*-ratio products with 5-chloro-1-pentanol as the acceptor, suggesting the stable and reliable catalytic ability of Sc(OTf)_3_ (Entries 1, 6, and 7, Table 4). Furthermore, glycosylation of donor **5** or **1** with different acceptors, including 5-chloro-1-pentanol, 3-bromo-1-propanol, or 4-hydroxyphenylboronic acid pinacol ester, also furnished expected *β*-selective products with yields from 83% to 91%. All of the structures were confirmed by NMR. Taken together, the results in Table 4 show that Sc(OTf)_3_ could be used as a more effective catalyst to promote the glycosylation of various donors and acceptors with high *β*-selectivity.

**TABLE 4:** Sc(OTf)_3_ (50 mol%) promoted glycosylation in refluxing 1,2-C_2_H_4_Cl_2_.

Entry	Donor	Acceptor	Product	Yield (%)^a^	*α*:*β* ratio^b^
1	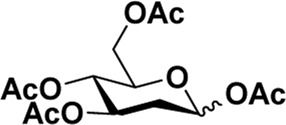	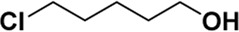	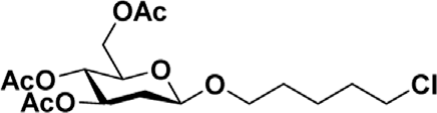	82	0:1
	**4**	**3**	**10**		
2	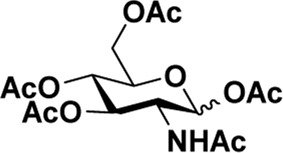	**3**	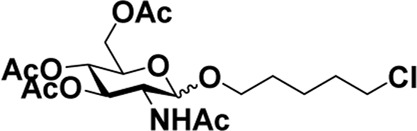	91	1:5
	**5**		**11**		
3	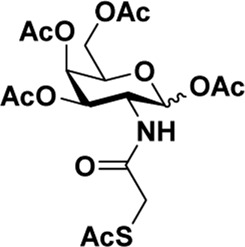	**3**	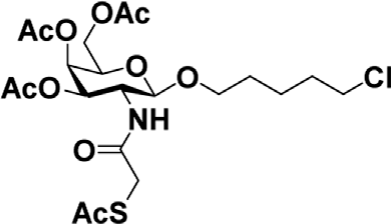	78	0:1
	**6**		**12**		
4	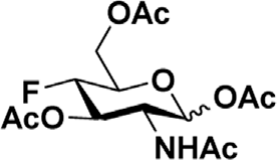	**3**	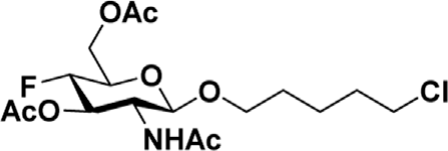	84	0:1
	**7**		**13**		
5	**5**	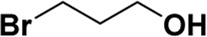	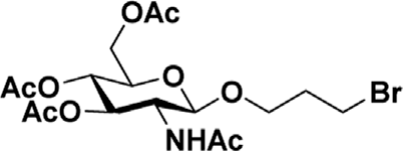	80	0:1
		**8**	**14**		
6	**5**	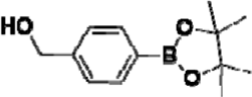	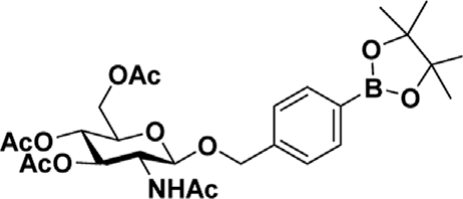	83	0:1
		**9**	**15**		
7	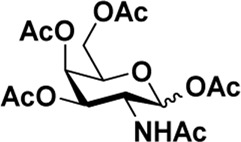	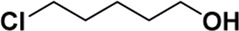	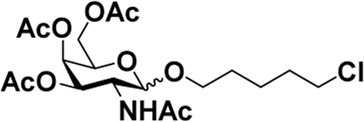	86	1:9
	**1**	**3**	**2**		

^a^ Yield after silica gel chromatography.

^b^ Observed by a TLC plate.

As mentioned previously, *α*-GalNAc glycosides are vital precursors for wide application *in vitro*, for example, GalNAc-*α*-ProN_3_ is used as the acceptor for the synthesis of fluorinated T antigens (Yan et al., 2013), and GalNAc-*α*-Bn analogs are employed as precursors to profile and amplify the mucin-type *O*-glycome in living cells (Zhang et al., 2019). With this in mind, we next investigated the *α*-selectivity of GalNAc-ylation with benzyl alcohol and substituted aliphatic alcohol as the acceptor under similar conditions as mentioned earlier (Table 5). We were pleased to find that all the glycosylation products conducted in the presence of Hf(OTf)_4_ exhibited high *α*-selectivity for various acceptors. Among these successes, glycosylation with acceptors of benzyl alcohol or 4-nitrophenol showed exclusive α-products in yields of 88% and 65%, respectively. In addition, aliphatic alcohols, including 5-chloro-1-pentanol and 3-bromo-1-propanol, also afforded α-selective glycosylation products with an α/β ratio of more than 6/1. Finally, Ac_4_GlcNAc **5**, as a common glycosyl donor, was also employed to evaluate the effectiveness of this method, and the results exhibited satisfactory α:β selectivity of 9.4:1 in a yield of 85%, indicating an extensive prospect of application. Our findings, in this study, raise the possibility that this strategy allows the preparation of more interested *α*-GalNAc-containing derivatives for further biological investigation.

**TABLE 5:** Hf(OTf)_4_ (50 mol%) promoted glycosylation in refluxing 1,2-C_2_H_4_Cl_2_.

Entry	Donor	Acceptor	Product	Yield (%)^a^	*α*:*β* ratio
1	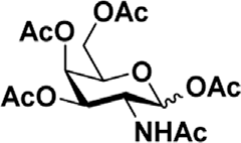	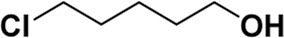	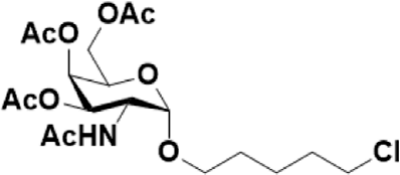	85	>10:1
	**1**	**3**	**18**		
2	**1**	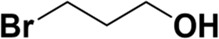	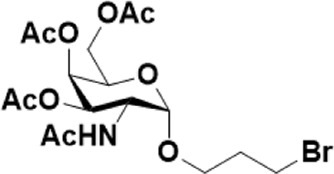	82	6:1
		**8**	**19**		
3	**1**	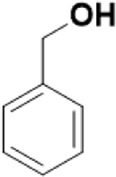	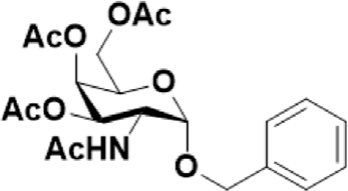	88	1:0
		**16**	**20**		
4	**1**	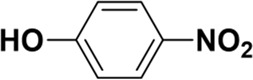	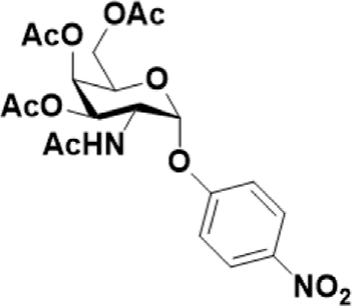	65	1:0
		**17**	**21**		
5	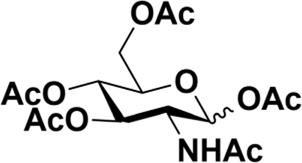	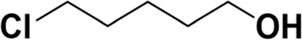	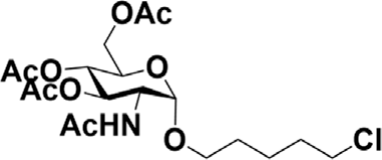	85	9.4:1
	**5**	**3**	**22**		

^a^ Yield after silica gel chromatography.

### Synthesis of GalNAc-naphthalimide-azide for detecting H_2_S in hepatocyte cells

**FIGURE 1:**
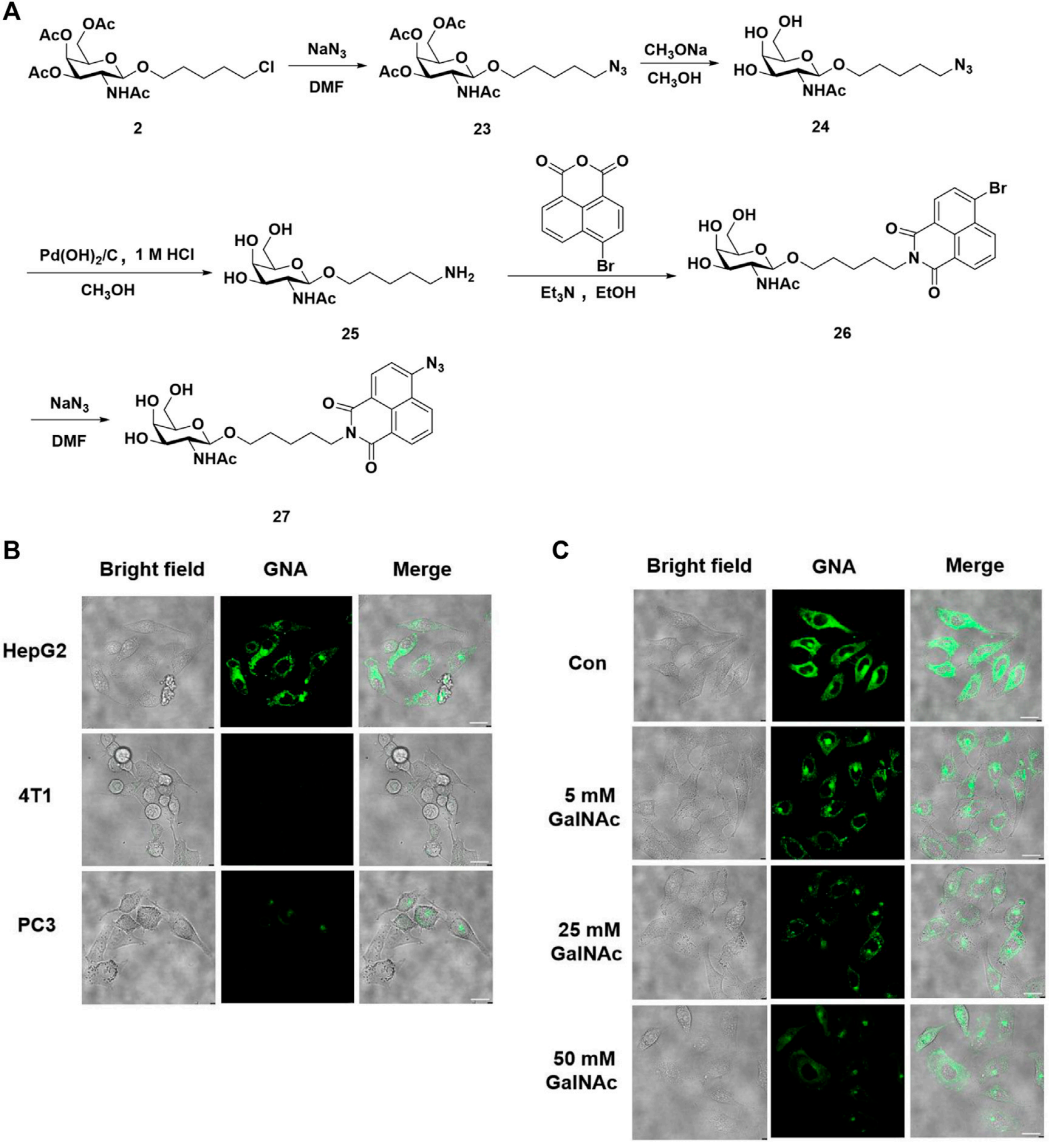
GalNAc-naphthalimide-azide (GNA) is a specific probe for H_2_S detection in hepatocyte cells. **(A)** Synthesis of GNA. **(B)** Fluorescence imaging of H_2_S in three kinds of mammalian cells with GNA **(C)** GNA uptaken into HepG2 cells was ASGR dependent (scale bar = 20 μm).

Majority of evidence has reported that glycosylation could lead to the improvement of solubility, targeting ability for specific cells, and reduced cytotoxicity. The strategy of incorporation of various carbohydrate moieties, including galactose, sialic acid, and glucose into natural products or peptides, has been proved to be an effective strategy to improve the bio-availability and anti-tumor activity (Pohl et al., 1995; Moradi et al., 2016; Kabotso et al., 2020). Moreover, our group has previously reported a series of drugs based on the naphthalimide structures, which was notorious for poor water solubility, although it was widely used as a drug design and fluorescent probe (Ma et al., 2020; Ma et al., 2021). Hydrogen sulfide, an important signaling molecule in living organisms, exerts multiple regulatory functions, such as stimulating vasodilation and maintaining redox homeostasis. A series of small-molecule probes have been explored for real-time and accurate detection of endogenous H_2_S *in vivo* (Li et al., 2019; Zhu et al., 2020). Based on previous success, we herein prepared a novel probe termed GalNAc-naphthalimide-azide (GNA) that is a selective fluorescence turn-on of H_2_S in HepG2 cells (Figure 1A). For the synthesis of GNA, the chloride residue in intermediate **2** was first substituted with sodium azide, followed by globe deacetylation, which was then reduced in the presence of Pd(OH)_2_/C to generate GalNAc-terminated amine **25**. The resultant directly reacted with 4-bromide-naphthalic anhydride to afford **26**, which was then substituted with sodium azide to replace the bromide on the naphthalimide to afford the desired product GNA. With GNA in hand, we next evaluated the selectivity of the developed probes by incubation of 10 μM GNA with three kinds of mammalian cells, specifically, human hepatocyte HepG2 cells, mouse-derived breast cancer 4T1 cells, and human prostate cancer PC3 cells. Each cell line was cocultured with GNA for 1 h and imaged, and the results demonstrated that GNA could be selectively taken up into HepG2 cells other than 4T1 and PC3 cells, revealing the high recognition and selectivity of GalNAc residues to hepatocyte cells (Figure 1B). To further confirm this specific turn-on fluorescence in HepG2 cells through ASGR-R-mediated endocytosis, various concentrations of GalNAc, from 5 mM to 50 mM, were added to compete with the up-take of GNA in HepG2 cells. The images in Figure 1C indicated that GalNAc competed with GNA for the entrance into HepG2 cells with increasing concentrations, leading to significantly reduced signals and providing confirmation that the result of GNA imaging of H_2_S was conducted with ASGR-R. Taken together, we believe that the fluorescent probe of GNA developed here is a potential specific probe for the detection of the levels of H_2_S in hepatocyte cells.

## Conclusion

In this research, a panel of rare earth metal triflates has been employed to construct GalNAc-containing glycosides. Among them, Sc(OTf)_3_ is a promising promoter for the synthesis of GalNAc *β*-glycosides in refluxing 1,2-C_2_H_4_Cl_2_ with satisfying yields, while Hf(OTf)_4_ is more effective and preferred for the *α*-configuration. A potential explanation for these selectivities may be that the *β*-selectivity of Sc(OTf)_3_ probably depends on the kinetic stability, while the products conducted with Hf(OTf)_4_, to some extent, are thermodynamically dependent. In addition, other glycosyl donors and acceptors are also used to convince the reliability of the methodology. Finally, to evaluate the applicability of this strategy for the synthesis of the GalNAc conjugate, a selective turn-on fluorescent probe based on GalNAc and 4-azide-naphthalimide is synthesized and evaluated for specific detection of H_2_S levels in HepG2 cells, which exhibits high selectivity in HepG2 cells and with negligible fluorescent signals in other cell lines. Overall, we believe this protocol offers a convenient approach for the synthesis of *α*- and *β*-glycosides of GalNAc that will be of high value to carbohydrate biological research.

## Data Availability

The original contributions presented in the study are included in the article/Supplementary Material; further inquiries can be directed to the corresponding authors.
