# Supplementary material for: Selective synthesis of α- and β-glycosides of N-acetyl galactosamine using rare earth metal triflates
Source: Front Chem. 2022 Oct 26;10:1029911. doi: 10.3389/fchem.2022.1029911 (PMC9644022; doi:10.3389/fchem.2022.1029911)
Supplement: Supplementary file 1 [file DataSheet1.pdf]

Supporting Information

Selective synthesis of *α*- and *β*-Glycosides of *N*-Acetyl galactosamine using rare earth metal triflates

Jiajia Wang [a,#], Wei Zhang [b,#], Wei Cao [a], Kang Liu [a], Shihao Su [b], Jing Ma [b,*], Xia Li [a,*]

[a] *Joint National Laboratory for Antibody Drug Engineering, the First Affiliated Hospital of Henan University, Henan University, Kaifeng, 475000, China;*

[b] *School of Pharmacy, Academy for Advanced Interdisciplinary Studies, Institute of Chemical Biology, Henan University, Kaifeng, Henan, China*

Table of Contents

Result of glycosylations S1

^{1}H NMR and ^{13}C NMR Characterization for compounds. S3

^{1}H NMR and ^{13}C NMR spectra for compounds. S7

^{1}H NMR and ^{13}C NMR for compound 2. .... S7

^{1}H NMR and ^{13}C NMR for compound 10. .... S8

^{1}H NMR and ^{13}C NMR for compound 11. .... S9

^{1}H NMR and ^{13}C NMR for compound 12. .... S10

^{1}H NMR and ^{13}C NMR for compound 13. .... S11

^{1}H NMR and ^{13}C NMR for compound 14. .... S12

^{1}H NMR and ^{13}C NMR for compound 15. .... S12

^{1}H NMR and ^{13}C NMR for compound 18. .... S13

^{1}H NMR and ^{13}C NMR for compound 19. .... S14

^{1}H NMR and ^{13}C NMR for compound 20. .... S15

^{1}H NMR and ^{13}C NMR for compound 21. .... S16

^{1}H NMR and ^{13}C NMR for compound 22..S16

^{1}H NMR and ^{13}C NMR for compound 27. .... S19

Results of glycosylations.

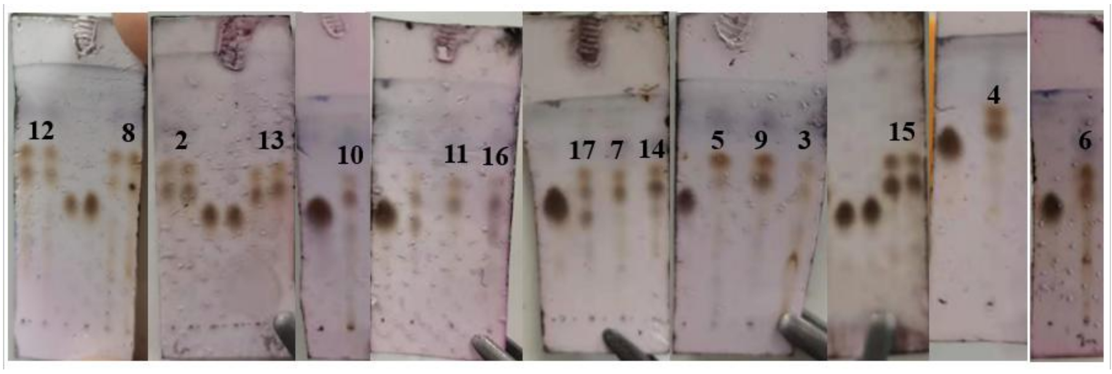

Figure S1. Results of glycosylations are shown on TLCs.

Entry	T/°C[a]	Solvent	Cat (50 mol %)	Percentage (α: β)	Time (h)	Conversion[b]
1	50	1,2-$C_2H_4Cl_2$	$Cu(OTf)_2$	10/10	29	20
2	90	1,2-$C_2H_4Cl_2$	$Cu(OTf)_2$	40/60	6	100
3	90	1,2-$C_2H_4Cl_2$	$Al(OTf)_3$	40/60	12	100
4	90	1,2-$C_2H_4Cl_2$	$In(OTf)_3$	40/60	15	100
5	90	1,2-$C_2H_4Cl_2$	$Hf(OTf)_4$	90/10	12	100
6	90	1,2-$C_2H_4Cl_2$	$Zn(OTf)_2$	50/50	12	100
7	90	1,2-$C_2H_4Cl_2$	$Gd(OTf)_3$	40/60	24	100
8	90	1,2-$C_2H_4Cl_2$	AgOTf	50/50	12	100
9	90	1,2-$C_2H_4Cl_2$	$Er(OTf)_3$	40/60	12	100
10	90	1,2-$C_2H_4Cl_2$	$Sc(OTf)_3$	10/90	12	100
11	90	1,2-$C_2H_4Cl_2$	$Ce(OTf)_3$	40/60	24	100
12	90	1,2-$C_2H_4Cl_2$	$Fe(OTf)_3$	50/50	12	100
13	90	1,2-$C_2H_4Cl_2$	$Yb(OTf)_3$	30/70	6	100
14	90	1,2-$C_2H_4Cl_2$	NaOTf	20/60	24	80
15	90	1,2-$C_2H_4Cl_2$	$Y(OTf)_3$	40/50	25	90
16	90	1,2-$C_2H_4Cl_2$	$La(OTf)_3$	20/60	24	80
17	90	1,2-$C_2H_4Cl_2$	$Sa(OTf)_3$	30/40	30	70

[a] Oil bath temperature. [b] Shown on TLCs.

Table S1 Results of glycosylations according to Figure S1

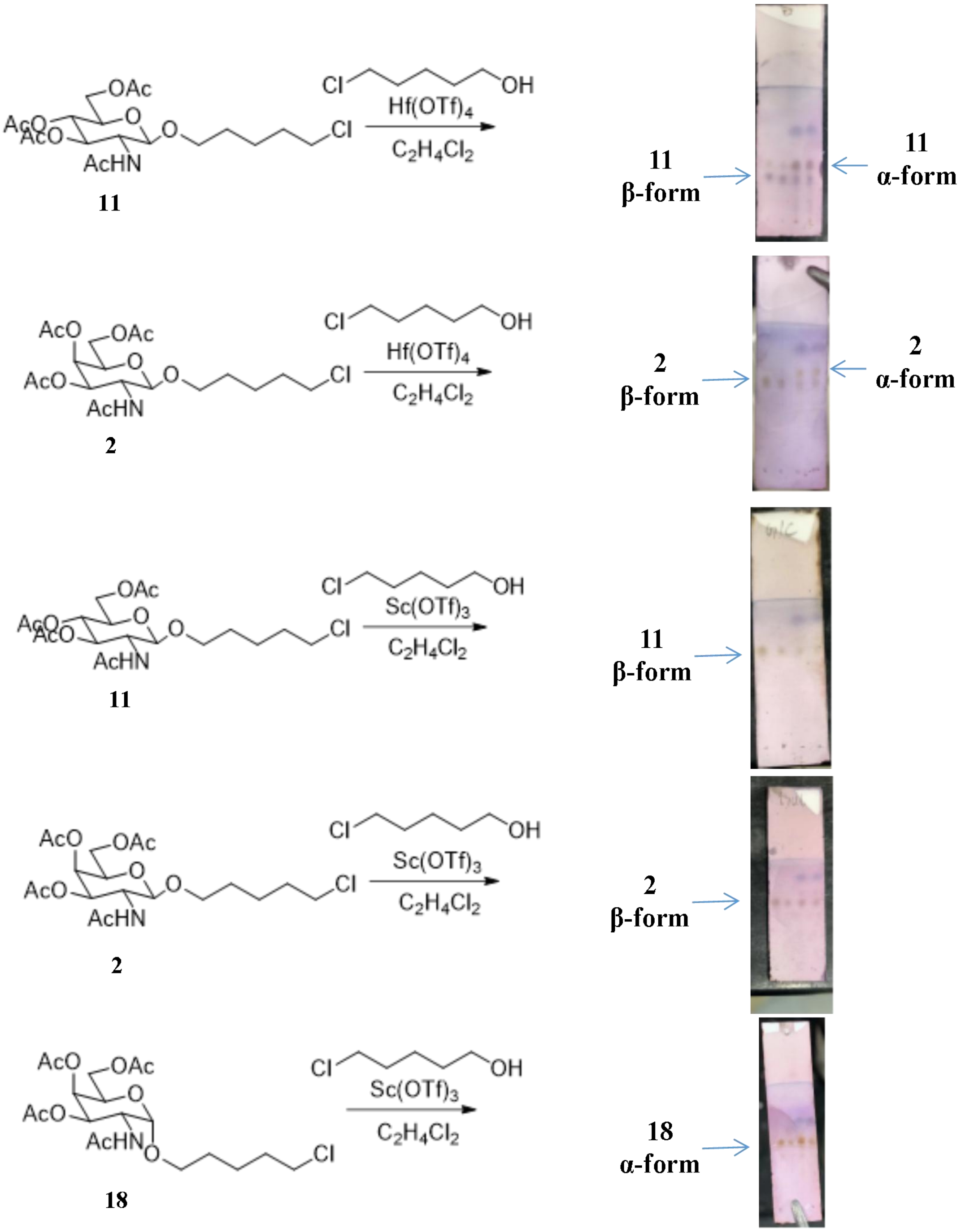

Figure S2. The reversibility of glycosylation products conducted in the presence of Sc(OTf)3 or Hf(OTf)4 to demonstrate the selectivity. (The two spots on the left were starting material, and the reaction were shown on the right)

^{1}H NMR and ^{13}C NMR Characterization for compounds.

Compound 2: 2-Acetamido-1,3,4,6-tetrakis-(O-acetyl)-2-deoxy-D-galactospyranose **1** (500 mg, 1.28 mmol) and indicated rare earth metal trifluoromethane sulfonate (0.64 mmol) were dissolved in the $C_2H_4Cl_2$ (20 mL) then the 5-chloropentan-1-ol (0.46 mL, 3.85 mmol) was added. The reaction mixture was stirred at 90 ° C for the indicated time, which was detected by TLC until no more generation of products. The solvent was then evaporated under reduced pressure and the crude mixture purified through silica gel column (PE: EA 1:1.5) to yield compound **2** as a white solid. ^{1}H NMR (300 MHz, $CDCl_3$) δ 5.88 (d, *J* = 8.5 Hz, 1H), 5.38 (s, 2H), 5.34 (d, *J* = 3.4 Hz, 1H), 4.75 (d, *J* = 8.4 Hz, 1H), 4.17 (dd, *J* = 6.7, 3.8 Hz, 3H), 4.03 – 3.85 (m, 4H), 3.56 (t, *J* = 6.6 Hz, 4H), 2.17 (d, *J* = 3.4 Hz, 3H), 2.07 (s, 4H), 2.03 (d, *J* = 3.3 Hz, 3H), 1.99 (s, 3H), 1.80 (p, *J* = 6.8 Hz, 3H), 1.63 (dt, *J* = 12.1, 6.7 Hz, 3H), 1.52 (dt, *J* = 12.7, 6.8 Hz, 3H), 1.28 (t, *J* = 7.1 Hz, 1H). ^{13}C NMR (75 MHz, $CDCl_3$) δ 170.87, 170.65, 170.44, 100.84, 70.56, 69.93, 69.61, 66.88, 61.62, 51.79, 45.09, 32.18, 28.67, 23.45, 23.32, 20.75.

Compound 10: 1,3,4,6-Tetra-O-acetyl-2-deoxy-α-D-glucopyranose **4** (500 mg, 1.51 mmol) and the Scandium trifluoromethane sulfonate (368 mg, 0.75 mmol) were dissolved in the $C_2H_4Cl_2$ (20 mL) then the 5-chloropentan-1-ol (0.54 mL, 4.52 mmol) was added. The reaction mixture was stirred for overnight at 90 ° C. The solvent was then evaporated under reduced pressure and the crude mixture purified through silica gel column (PE: EA 2:1) to yield compound 10 as a colorless oil. ^{1}H NMR (300 MHz, $CDCl_3$) δ 5.35 (ddd, *J* = 11.6, 9.4, 5.4 Hz, 1H), 5.04 (d, *J* = 9.8 Hz, 1H), 4.37 – 4.28 (m, 1H), 4.09 (dd, *J* = 12.2, 2.4 Hz, 1H), 3.98 (ddd, *J* = 10.0, 4.7, 2.3 Hz, 1H), 3.59 (t, *J* = 6.6 Hz, 2H), 3.42 (dt, *J* = 10.1, 6.4 Hz, 1H), 2.26 (ddd, *J* = 12.9, 5.4, 1.4 Hz, 1H), 2.16 – 2.01 (m, 9H), 1.92 – 1.78 (m, 3H), 1.71 – 1.50 (m, 5H), 1.38 – 1.27 (m, 1H). ^{13}C NMR (75 MHz, $CDCl_3$) δ 170.75, 170.24, 169.94, 96.98, 69.49, 69.16, 67.84, 67.50, 62.48, 44.86, 35.07, 32.33, 28.70, 23.62, 21.00, 20.79, 20.76.

Compound 11: **5** (500 mg, 1.28 mmol) and the Scandium trifluoromethane sulfonate 316 mg, 0.64 mmol) were dissolved in the $C_2H_4Cl_2$ (20 mL) then the 5-chloropentan-1-ol (0.46 mL, 3.85 mmol) was added. The reaction mixture was stirred for overnight at 90 ° C. The solvent was then evaporated under reduced pressure and the crude mixture purified through silica gel column (PE: EA 1:1.5) to yield compound **11** as a white solid. ^{1}H NMR (400 MHz, $CDCl_3$) δ 5.52 (d, *J* = 8.7 Hz, 1H), 5.30 (dd, *J* = 10.5, 9.4 Hz, 1H), 5.07 (t, *J* = 9.6 Hz, 1H), 4.68 (d, *J* = 8.3 Hz, 1H), 4.26 (dd, *J* = 12.2, 4.8 Hz, 1H), 4.14 (dd, *J* = 12.2, 2.4 Hz, 1H), 3.97 – 3.78 (m, 2H), 3.70 (ddd, *J* = 10.0, 4.7, 2.5 Hz, 1H), 3.50 (ddd, *J* = 13.1, 11.4, 6.7 Hz, 3H), 2.09 (s, 3H), 2.03 (s, 2H), 2.03 (s, 2H), 1.96 (s, 3H), 1.78 (dq, *J* = 13.5, 6.7 Hz, 2H), 1.63 – 1.58 (m, 2H), 1.53 – 1.43 (m, 2H). ^{13}C NMR (100 MHz, $CDCl_3$) δ 170.93, 170.75, 170.18, 169.44, 100.72, 72.33, 71.83, 69.43, 68.67, 62.16, 54.88, 45.02, 32.17, 28.66, 23.37, 23.33, 20.78, 20.71, 20.65.

Compound 12: **7** (500mg, 1.08 mmol) and the Scandium trifluoromethane sulfonate (266 mg, 0.54 mmol) were dissolved in the $C_2H_4Cl_2$ (20 mL) then the 5-chloropentan-1-ol (0.39 mL, 3.24 mmol) was added. The reaction mixture was stirred for overnight at 90 ° C. The solvent was then evaporated under reduced pressure and the crude mixture purified through silica gel column (PE: EA 2:1) to yield compound **12** as a yellow oil. ^{1}H NMR (300 MHz, $CDCl_3$) δ 6.35 (d, *J* = 8.9 Hz, 1H), 5.33 (d, *J* = 3.4 Hz, 1H), 5.21 (dd, *J* = 11.2, 3.4 Hz, 1H), 4.65 (d, *J* = 8.4 Hz, 1H), 4.15 (dd, *J* = 6.7, 4.3 Hz, 2H), 3.90 (dp, *J* = 9.3, 6.1, 5.6 Hz, 2H), 3.61 – 3.40 (m, 3H), 3.29 (t, *J* = 6.8 Hz, 1H), 2.41 (s, 3H), 2.15 (s, 3H), 2.05 (s, 3H), 2.00 (s, 3H), 1.60 (d, *J* = 6.9 Hz, 2H), 1.37 – 1.21 (m, 4H).^{13}C NMR (75 MHz, $CDCl_3$) δ 194.45, 170.32, 169.68, 169.33, 168.04, 99.33, 67.89, 67.15, 66.26, 64.41, 61.93, 48.96, 44.77, 32.97, 32.15, 30.18, 28.47, 23.41, 20.72, 20.64, 20.53.

Compound 13: **7** (500 mg, 1.43 mmol) and the Scandium trifluoromethane sulfonate (351 mg, 0.71 mmol) were dissolved in the $C_2H_4Cl_2$ (20 mL) then the 5-chloropentan-1-ol (0.52 mL, 4.29 mmol) was added. The reaction mixture was stirred for overnight at 90 ° C. The solvent was then evaporated under reduced pressure and the crude mixture purified through silica gel column (PE: EA 1:2) to yield compound **13** as a colorless oil. ^{1}H NMR (300 MHz, $CDCl_3$) δ 5.85 (d, *J* = 9.4 Hz, 1H), 5.31 (ddd, *J* = 14.2, 10.8, 9.0 Hz, 1H), 4.79 (t, *J* = 3.3 Hz, 1H), 4.58 (t, *J* = 9.4 Hz, 1H), 4.40 (dd, *J* = 15.5, 5.4 Hz, 2H), 4.32 – 4.19 (m, 2H), 4.05 – 3.93 (m, 1H), 3.71 (dt, *J* = 9.9, 6.4 Hz, 1H), 3.56 (t, *J* = 6.5 Hz, 2H), 3.51 – 3.38 (m, 2H), 2.10 (s, 3H), 2.09 (s, 2H), 1.96 (s, 4H), 1.80 (dd, *J* = 14.4, 6.8 Hz, 2H), 1.64 (dd, *J* = 13.6, 6.7 Hz, 2H), 1.53 (ddd, *J* = 12.8, 5.7, 2.9 Hz, 2H). ^{13}C NMR (75 MHz, $CDCl_3$) δ 171.25, 170.57, 170.13, 97.06, 87.89, 85.42, 71.36, 71.11, 68.15, 67.40, 67.09, 62.06, 51.74, 51.65, 44.81, 32.08, 28.48, 23.40, 23.12, 20.82, 20.73.

Compound 14: **5** (500 mg, 1.28 mmol) and the Scandium trifluoromethane sulfonate (316 mg, 0.64 mmol) were dissolved in the $C_2H_4Cl_2$ (20 mL) then the 3-bromopropan-1-ol (0.35 mL, 3.85 mmol) was added. The reaction mixture was stirred for overnight at 90 ° C. The solvent was then evaporated under reduced pressure and the crude mixture purified through silica gel column (PE: EA 1:1.5) to yield compound **14** as a white solid. ^{1}H NMR (300 MHz, $CDCl_3$) δ 6.61 (d, *J* = 8.3 Hz, 1H), 5.40 – 5.25 (m, 1H), 5.07 (t, *J* = 9.7 Hz, 1H), 4.70 (d, *J* = 8.4 Hz, 1H), 4.28 (dd, *J* = 12.3, 4.9 Hz, 1H), 4.15 (dd, *J* = 12.2, 2.1 Hz, 1H), 4.06 – 3.88 (m, 2H), 3.79 (ddd, *J* = 9.8, 4.6, 2.2 Hz, 1H), 3.69 (dt, *J* = 9.5, 4.5 Hz, 1H), 3.52 (dt, *J* = 8.1, 3.9 Hz, 3H), 2.10 (s, 3H), 2.04 (s, 4H), 2.03 (s, 3H), 1.99 (s, 3H). ^{13}C NMR (75 MHz, $CDCl_3$) δ 170.74, 170.61, 169.40, 101.25, 72.41, 71.60, 68.79, 67.21, 62.21, 54.25, 32.18, 30.53, 23.26, 20.77, 20.71, 20.64.

Compound 15: **5** (500 mg, 1.28 mmol) and the Scandium trifluoromethane sulfonate (316 mg, 0.64 mmol) were dissolved in the $C_2H_4Cl_2$ (20 mL) then the 4-Methoxybenzeneboronic acid, pinacol ester (900 mg, 3.85 mmol) was added. The reaction mixture was stirred for overnight at 90 ° C. The solvent was then evaporated under reduced pressure and the crude mixture purified through silica gel column (PE: EA = 3:1) to yield compound **15** as a white solid. ^{1}H NMR (300 MHz, $CDCl_3$) δ 7.82 (d, *J* = 7.9 Hz, 2H), 7.37 – 7.30 (m, 3H), 5.54 (d, *J* = 9.0 Hz, 1H), 5.27 – 5.04 (m, 2H), 4.94 (d, *J* = 12.6 Hz, 1H), 4.73 – 4.53 (m, 2H), 4.30 (dd, *J* = 12.3, 4.7 Hz, 1H), 4.19 (dd, *J* = 12.3, 2.4 Hz, 1H), 4.12 – 3.95 (m, 1H), 3.68 (ddd, *J* = 9.4, 4.6, 2.4 Hz, 1H), 2.14 (s, 4H), 2.04 (s, 7H), 1.95 (s, 3H), 1.38 (s, 13H). ^{13}C NMR (75 MHz, $CDCl_3$) δ 171.01, 170.80, 170.23, 169.41, 139.95, 134.97, 127.27, 99.35, 83.93, 72.49, 71.89, 70.47, 68.56, 62.14, 54.41, 24.89, 23.33, 20.81, 20.71, 20.65.

Compound 18: **1** (500 mg, 1.28 mmol) and Hafnium the trifluoromethanesulphonate (316 mg, 0.64 mmol) were dissolved in the $C_2H_4Cl_2$ (20 mL) then the 5-chloropentan-1-ol (0.46 mL, 3.85 mmol) was added. The reaction mixture was stirred for overnight at 90 ° C. The solvent was then evaporated under reduced pressure and the crude mixture purified through silica gel column (PE: EA 1:1.5) to yield compound **18** as a white solid. ^{1}H NMR (400 MHz, $CDCl_3$) δ 5.62 (d, *J* = 9.7 Hz, 1H), 5.38 (d, *J* = 3.0 Hz, 1H), 5.16 (dd, *J* = 11.3, 3.3 Hz, 1H), 4.88 (d, *J* = 3.6 Hz, 1H), 4.58 (ddd, *J* = 11.2, 9.7, 3.7 Hz, 1H), 4.10 (dd, *J* = 6.4, 3.9 Hz, 2H), 3.71 (dt, *J* = 9.9, 6.4 Hz, 1H), 3.57 (t, *J* = 6.5 Hz, 2H), 3.50 – 3.42 (m, 1H), 2.17 (s, 3H), 2.05 (s, 4H), 2.00 (s, 3H), 1.97 (s, 3H), 1.88 – 1.76 (m, 3H), 1.65 (dd, *J* = 8.3, 5.9 Hz, 2H), 1.54 (q, *J* = 8.3 Hz, 2H).^{13}C NMR (100 MHz, $CDCl_3$) δ 171.02, 170.44, 170.38, 170.04, 97.65, 68.50, 68.04, 67.41, 66.73, 62.01, 47.86, 44.86, 32.11, 28.58, 23.49, 23.36, 20.79, 20.77, 20.72.

Compound 19: **1** (500 mg, 1.28 mmol) and the Scandium trifluoromethane sulfonate (316 mg, 0.64 mmol) were dissolved in the $C_2H_4Cl_2$ (20 mL) then the 3-bromopropan-1-ol (0.35 mL, 3.85 mmol) was added. The reaction mixture was stirred for overnight at 90 ° C. The solvent was then evaporated under

reduced pressure and the crude mixture purified through silica gel column (PE: EA 1:1.5) to yield compound **19** as a white solid. ^{1}H NMR (400 MHz, $CDCl_3$) δ 5.61 (d, *J* = 9.6 Hz, 1H), 5.38 (dd, *J* = 3.3, 1.2 Hz, 1H), 5.15 (dd, *J* = 11.4, 3.3 Hz, 1H), 4.91 (d, *J* = 3.6 Hz, 1H), 4.59 (ddd, *J* = 11.4, 9.5, 3.7 Hz, 1H), 4.11 (dd, *J* = 9.0, 6.4 Hz, 2H), 3.89 (dt, *J* = 10.0, 5.9 Hz, 1H), 3.52 (t, *J* = 6.1 Hz, 2H), 2.17 (d, *J* = 2.8 Hz, 4H), 2.06 (d, *J* = 6.4 Hz, 4H), 2.01 (s, 3H), 1.98 (s, 3H).^{13}C NMR (100 MHz, $CDCl_3$) δ 171.05, 170.48, 170.37, 170.11, 97.83, 68.34, 67.36, 66.94, 65.90, 61.99, 47.85, 31.85, 29.96, 23.34, 20.80, 20.76.

Compound 20: 1 (500 mg, 1.28 mmol) and the Hafnium trifluoromethanesulphonate (316 mg, 0.64 mmol) were dissolved in the $C_2H_4Cl_2$ (20 mL) then the benzyl alcohol (0.40 mL, 3.85 mmol) was added. The reaction mixture was stirred for overnight at 90 ° C. The solvent was then evaporated under reduced pressure and the crude mixture purified through silica gel column (PE: EA 2:1) to yield compound **20** as a white solid. ^{1}H NMR (400 MHz, $CDCl_3$) δ 7.43 – 7.28 (m, 5H), 5.38 (d, *J* = 3.2 Hz, 1H), 5.18 (dd, *J* = 11.3, 3.3 Hz, 1H), 4.98 (d, *J* = 3.7 Hz, 1H), 4.72 (d, *J* = 11.6 Hz, 1H), 4.59 (ddd, *J* = 11.6, 9.8, 3.7 Hz, 1H), 4.50 (d, *J* = 11.7 Hz, 1H), 4.22 (t, *J* = 6.6 Hz, 1H), 4.09 (qd, *J* = 11.2, 6.6 Hz, 2H), 2.16 (s, 3H), 2.06 (s, 3H), 1.99 (s, 3H), 1.91 (s, 3H). ^{13}C NMR (100 MHz, $CDCl_3$) δ 170.94, 170.44, 170.38, 170.09, 136.60, 128.72, 128.40, 128.25, 97.03, 70.16, 68.45, 67.40, 66.97, 61.94, 47.75, 23.26, 20.76, 20.73.

Compound 21: 1 (500 mg, 1.28 mmol) and the Hafnium trifluoromethanesulphonate (316 mg, 0.64 mmol) were dissolved in the $C_2H_4Cl_2$ (20 mL) then the 4-nitro-Benzenemethanol (0.41 mL, 3.85 mmol) was added. The reaction mixture was stirred for overnight at 90 ° C. The solvent was then evaporated under reduced pressure and the crude mixture purified through silica gel column (PE: EA 2:1) to yield compound **21** as a white solid. ^{1}H NMR (300 MHz, $CDCl_3$) δ 8.32 – 8.21 (m, 2H), 7.28 – 7.20 (m, 2H), 6.02 (d, *J* = 8.9 Hz, 1H), 5.74 (d, *J* = 3.5 Hz, 1H), 5.46 (dd, *J* = 10.9, 9.4 Hz, 1H), 5.26 (t, *J* = 9.8 Hz, 1H), 4.57 (ddd, *J* = 10.8, 8.9, 3.5 Hz, 1H), 4.24 (dd, *J* = 12.5, 4.6 Hz, 1H), 4.12 – 3.94 (m, 2H), 2.11 (s, 4H), 2.07 (d, *J* = 5.1 Hz, 7H), 2.00 (s, 3H), 1.33 – 1.26 (m, 1H). ^{13}C NMR (75 MHz, $CDCl_3$) δ 171.72, 170.50, 170.28, 169.23, 160.45, 143.14, 125.92, 116.42, 95.65, 70.53, 69.06, 67.53, 61.47, 51.95, 23.12, 20.77, 20.66, 20.59.

Compound 22: 5 (500 mg, 1.28 mmol) and Hafnium the trifluoromethanesulphonate (316 mg, 0.64 mmol) were dissolved in the $C_2H_4Cl_2$ (20 mL) then the 5-chloropentan-1-ol (0.46 mL, 3.85 mmol) was added. The reaction mixture was stirred for overnight at 90 ° C. The solvent was then evaporated under reduced pressure and the crude mixture purified through silica gel column (PE: EA 1:1.5) to yield compound **22** as a white solid. ^{1}H NMR (300 MHz, $CDCl_3$) δ 5.72 (d, *J* = 9.5 Hz, 1H), 5.10 (dt, *J* = 31.5, 9.6 Hz, 2H), 4.78 (d, *J* = 3.6 Hz, 1H), 4.28 (td, *J* = 10.1, 3.7 Hz, 1H), 4.18 (dd, *J* = 12.3, 4.6 Hz, 1H), 4.03 (dd, *J* = 12.3, 2.2 Hz, 1H), 3.89 (ddd, *J* = 9.9, 4.4, 2.3 Hz, 1H), 3.66 (dt, *J* = 9.9, 6.4 Hz, 1H), 3.52 (t, *J* = 6.4 Hz, 2H), 3.43 – 3.35 (m, 1H), 2.04 (s, 4H), 1.98 (s, 3H), 1.97 (s, 4H), 1.90 (s, 3H), 1.83 – 1.71 (m, 2H), 1.60 (dd, *J* = 13.5, 6.7 Hz, 2H), 1.54 – 1.43 (m, 2H). ^{13}C NMR (75 MHz, $CDCl_3$) δ 171.35, 170.64, 169.93, 169.30, 97.12, 71.31, 68.18, 68.01, 67.71, 62.04, 51.86, 44.79, 32.06, 28.48, 23.39, 23.13, 20.69, 20.57.

Compound 27:

(1) Compound **2** (1 g, 2.2 mmol) and sodium azido (288 mg, 4.4 mmol) were dissolved in anhydrous DMF (40 mL), the mixture was heated at 70 ℃ for 4 h. After remove the solvent under vacuum, the residue was extracted with EtOAc (300 mL) and subsequently washed with water (100 mL). Then the organic layer was dried over Na_2SO_4 and concentrated in vacuo.The resulting residue was purified by silica gel column chromatography to afford a a yellow-white solid **22** (900 mg,

88.6 %).

(2) The compound **23** (800 mg, 1.7 mmol) was dissolved in 50 mL anhydrous methanol and CH_3ONa was gradually added to adjust the solution pH to 9-10. After 6 hours reaction at room temperature, cation (H^+) resin was added to adjust the solution pH to neutral. The solution was filtered and concentrated in vacuo. the mixture was purified by silica gel column chromatography to afford a white solid **24** (491 mg, 85 %).

(3) A solution of compound **24** (450 mg, 1.4 mmol) in MeOH (30 mL) was treated with 10% $Pd(OH)_2$ (250 mg) as a catalyst and diluted 1 M HCl (0.5 mL). The reaction mixture was stirred under an atmosphere of H_2 at 30℃ overnight. The catalyst and the solvent were removed and the crude product **25** was directly used for next step (342 mg, 82.4 %).

(4) Compound **25** (300 mg, 1.0 mmol) and 4-bromide-Naphtalic (406 mg, 1.5 mmol) were dissolved in anhydrous EtOH (30 mL).Then Et_3N (0.3 mL, 2.0 mmol) was added and the reaction mixture was stirred under reflux overnight. Upon cooling to room temperature, the mixture was purified by silica gel column chromatography to afford a yellow solid **26** (385 mg, 69.6 %).

(5) Compound **26** (300 mg, 0.5 mmol) and sodium azido (69 mg, 1.0 mmol) were dissolved in anhydrous DMF (20 mL), the mixture was heated at 70 °C for 4h. After remove the solvent under vacuum, the residue was extracted with EtOAc (100 mL) and subsequently washed with water (30 mL). Then the organic layer was dried over Na_2SO_4 and concentrated in vacuo.The resulting residue was purified by silica gel column chromatography to afford a a yellow solid **27** (235 mg, 84.8 %). ^{1}H NMR (300 MHz, MeOD) δ 8.41 – 8.11 (m, 3H), 7.61 (s, 1H), 7.37 (d, *J* = 7.7 Hz, 1H), 4.69 (d, *J* = 8.8 Hz, 1H), 4.17 (dd, *J* = 8.8, 3.2 Hz, 1H), 3.76 (t, *J* = 6.1 Hz, 2H), 3.67 (d, *J* = 3.6 Hz, 1H), 3.37 (s, 1H), 3.33 (p, *J* = 1.7 Hz, 1H), 2.02 (d, *J* = 2.4 Hz, 3H), 1.67 (q, *J* = 7.6 Hz, 4H), 1.40 – 1.24 (m, 6H). ^{13}C NMR (75 MHz, MeOD) δ 171.70, 163.62, 163.17, 143.32, 131.44, 131.28, 128.28, 128.24, 126.48, 123.66, 121.86, 117.93, 114.68, 99.51, 73.67, 70.61, 69.25, 68.51, 61.36, 50.03, 39.89, 29.36, 28.98, 23.25, 21.53.

^{1}H NMR and ^{13}C NMR spectra for compounds.

^{1}H NMR and ^{13}C NMR for compound 2.

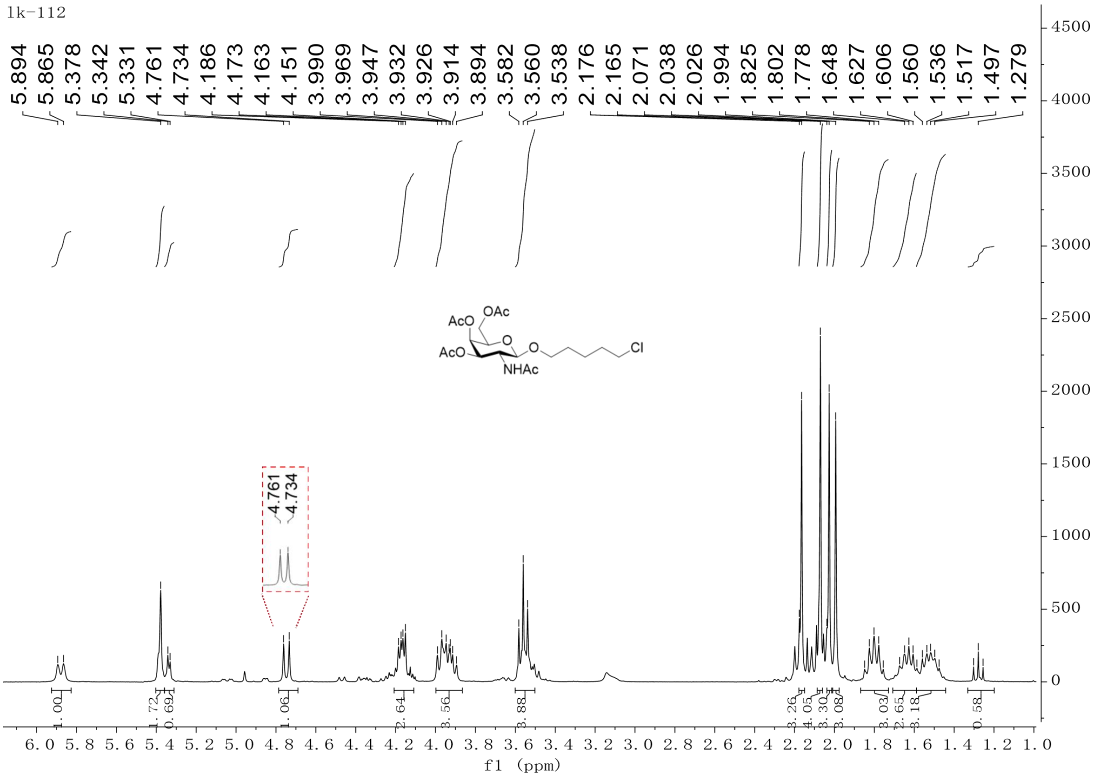

^{1}H NMR spectrum of compound **2**.

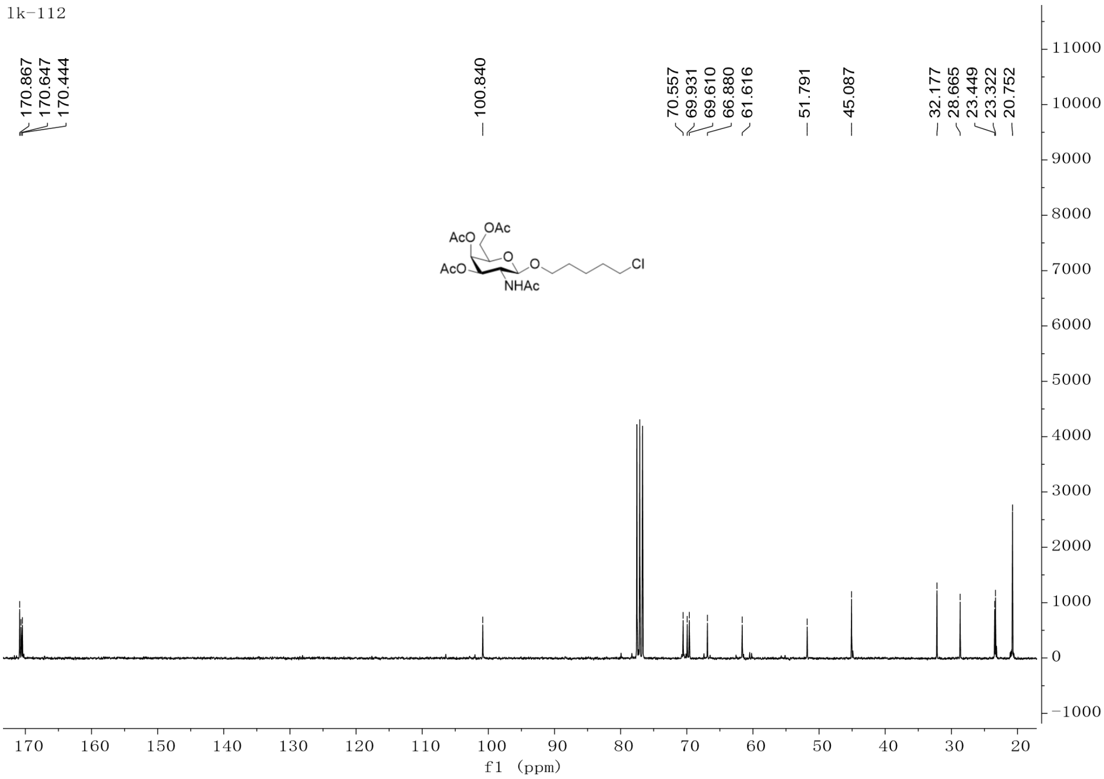

^{13}C NMR spectrum of compound **2**.

^{1}H NMR and ^{13}C NMR for compound 10.

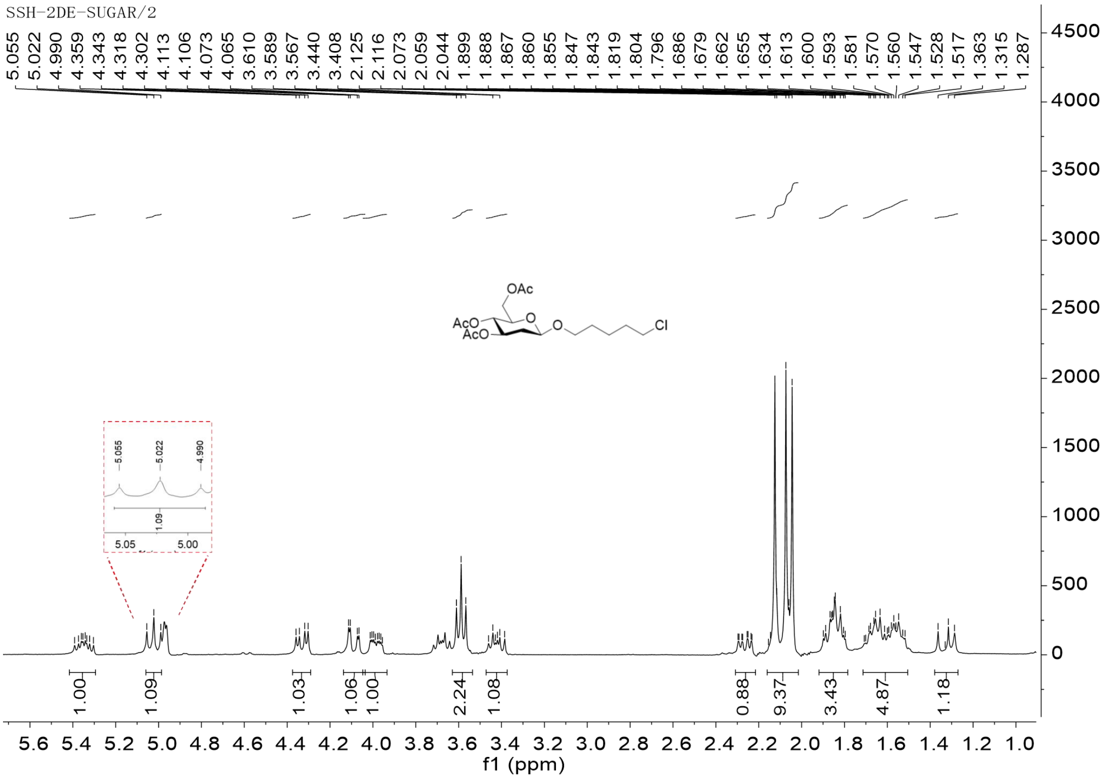

^{1}H NMR spectrum of compound **10.**

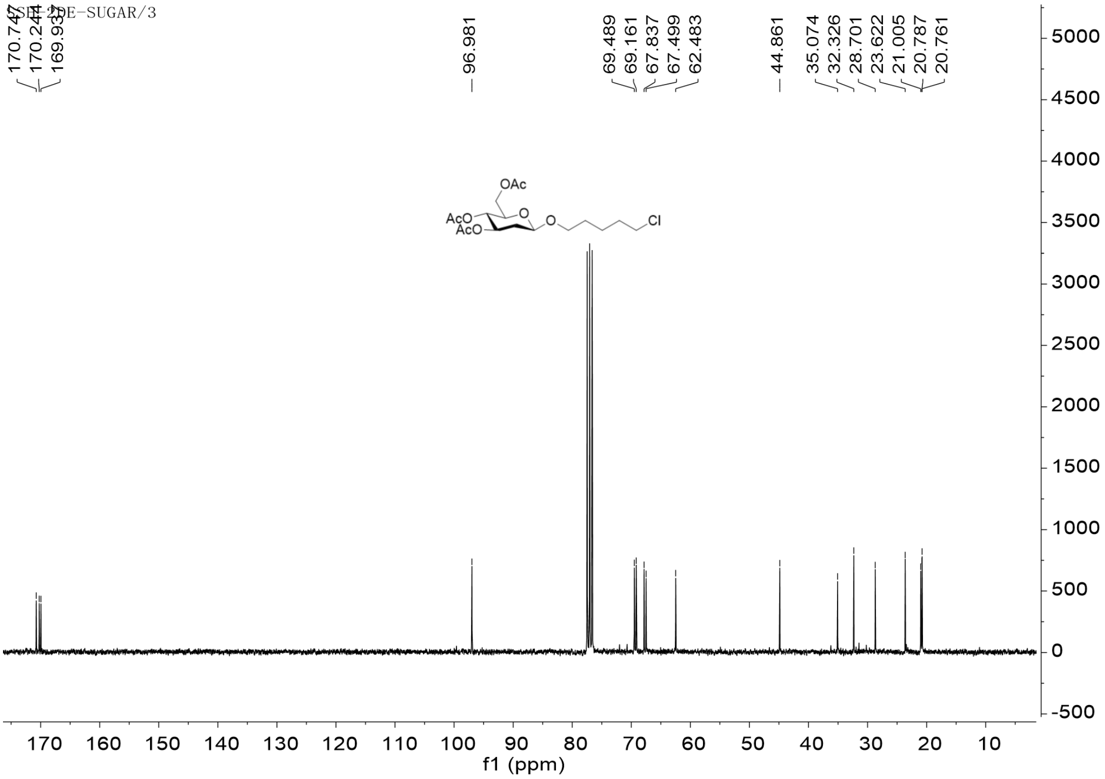

^{13}C NMR spectrum of compound **10**.

^{1}H NMR and ^{13}C NMR for compound 11.

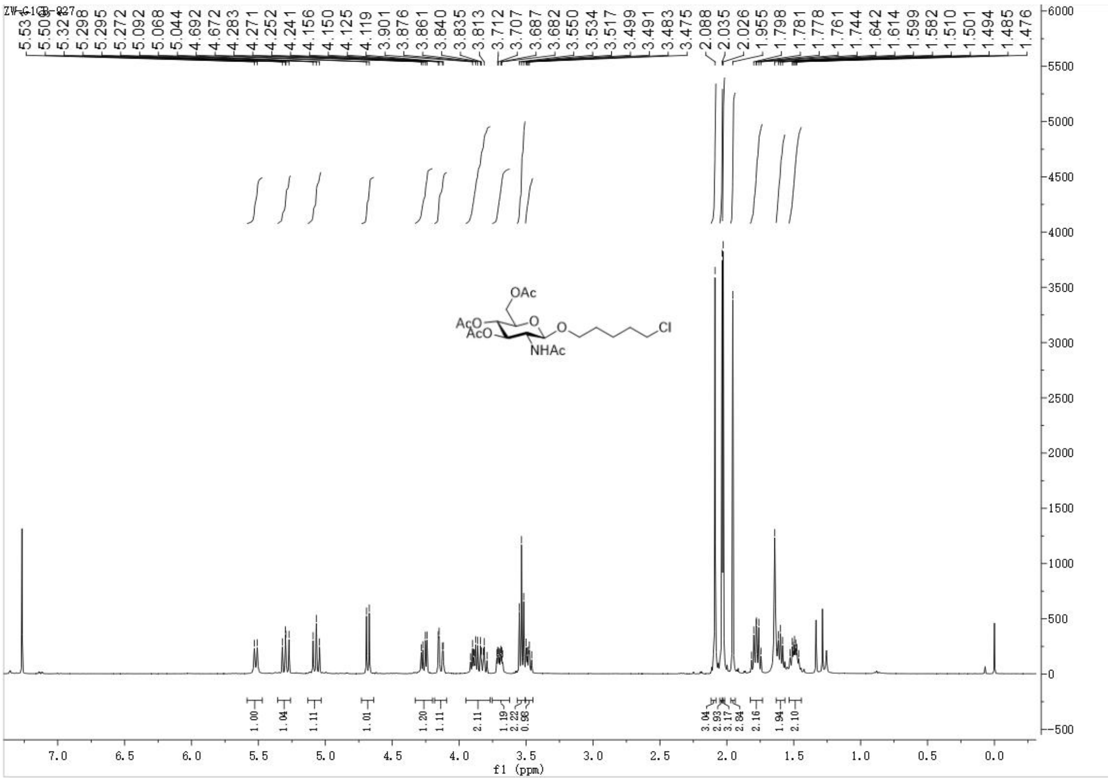

^{1}H NMR spectrum of compound **11**.

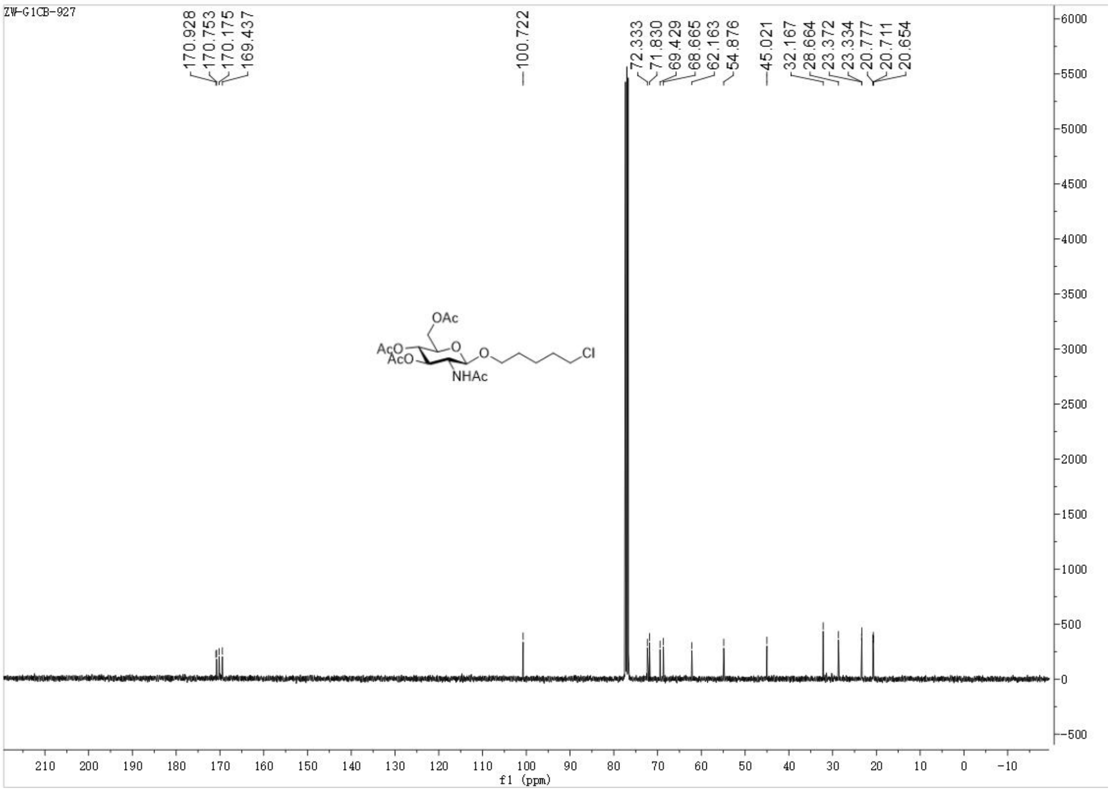

^{13}C NMR spectrum of compound **11**.

^{1}H NMR and ^{13}C NMR for compound 12.

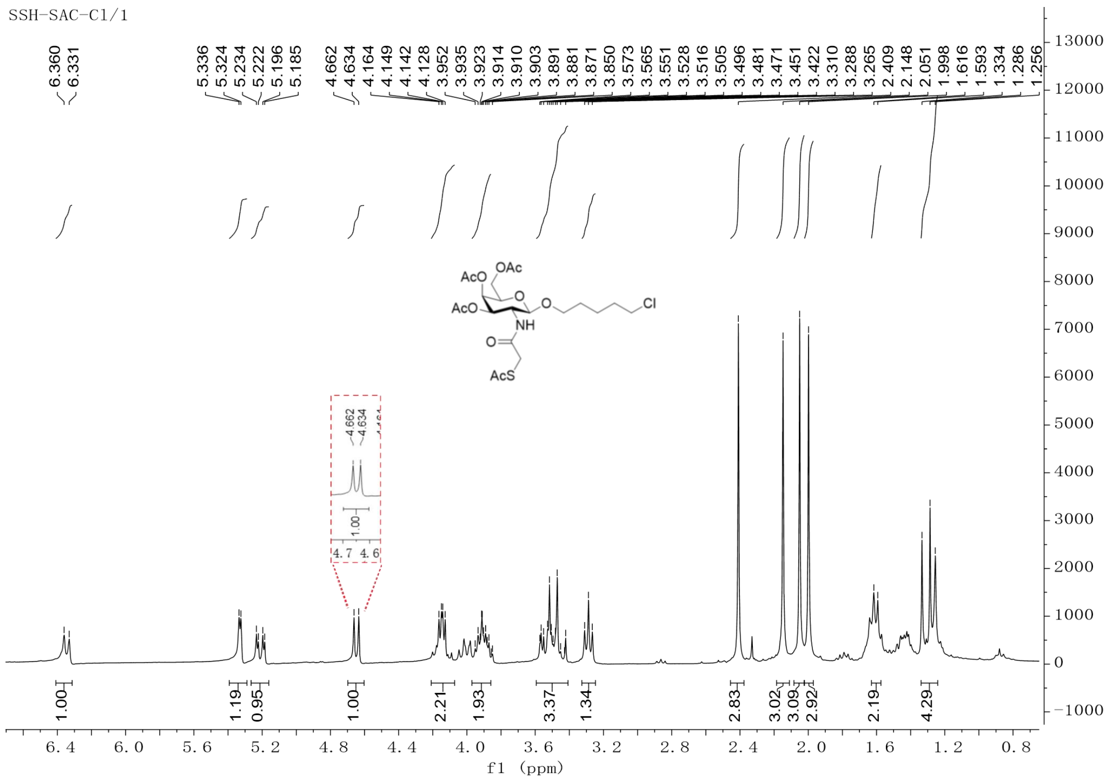

^{1}H NMR spectrum of compound **12.**

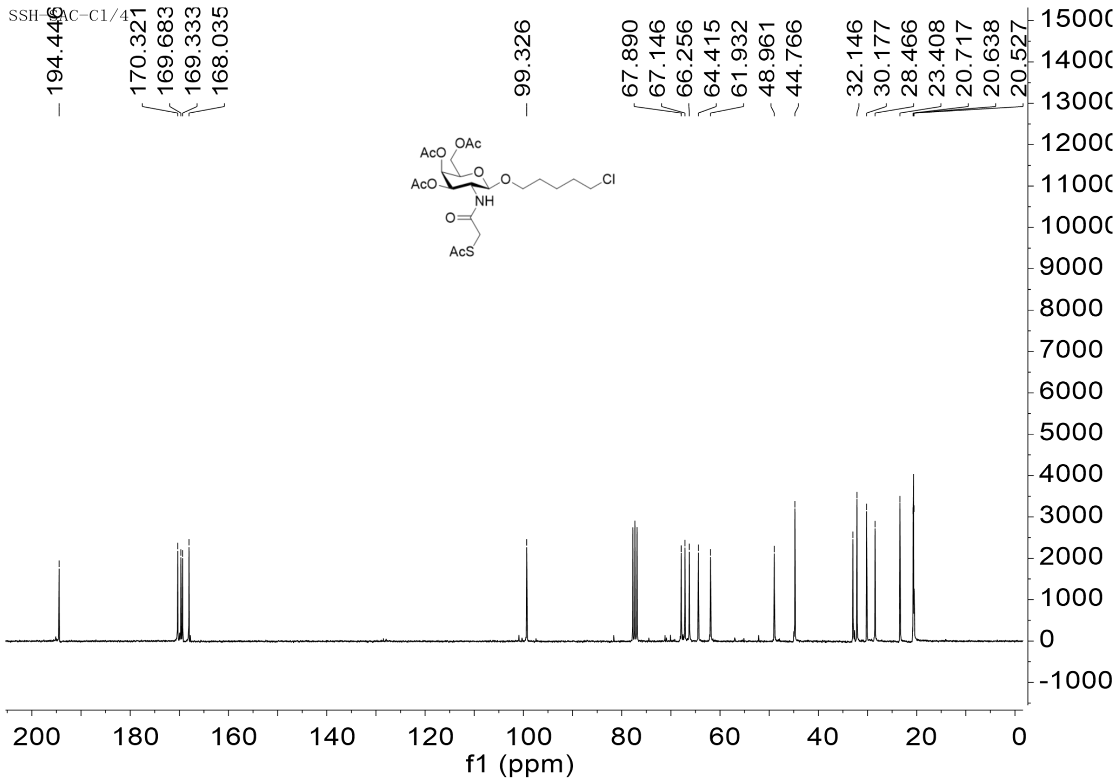

^{13}C NMR spectrum of compound **12**.

^{1}H NMR and ^{13}C NMR for compound 13.

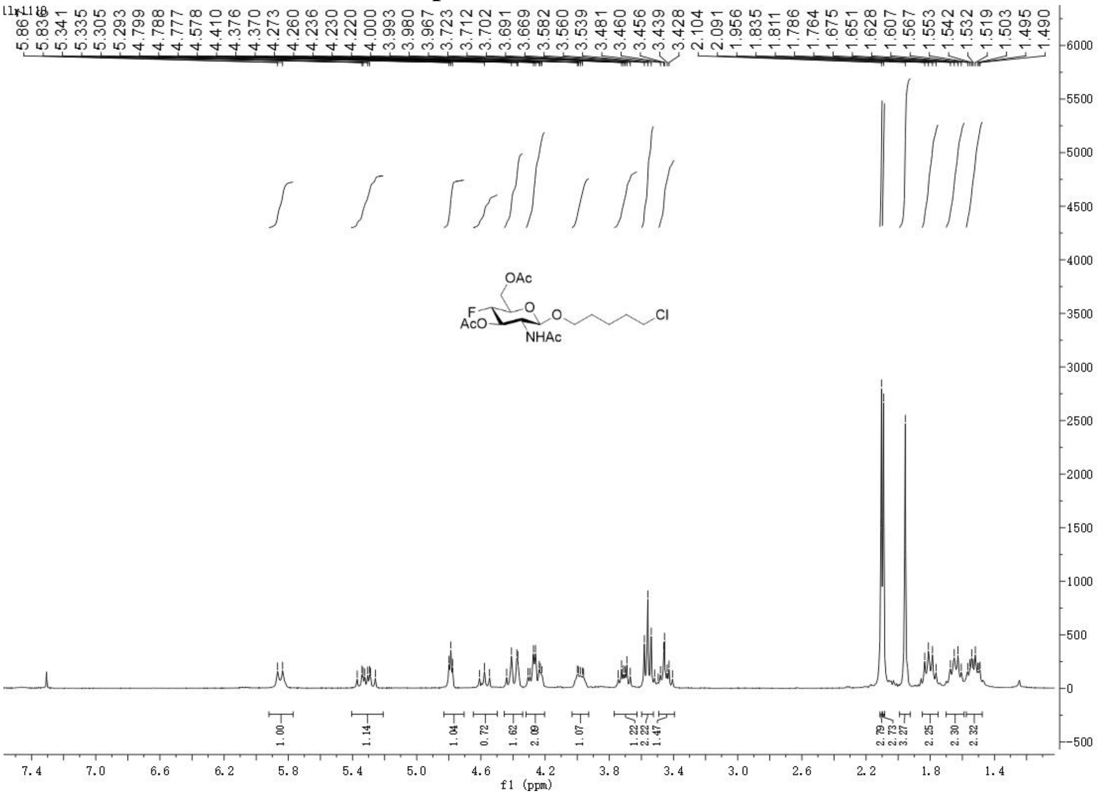

^{1}H NMR spectrum of compound **13**.

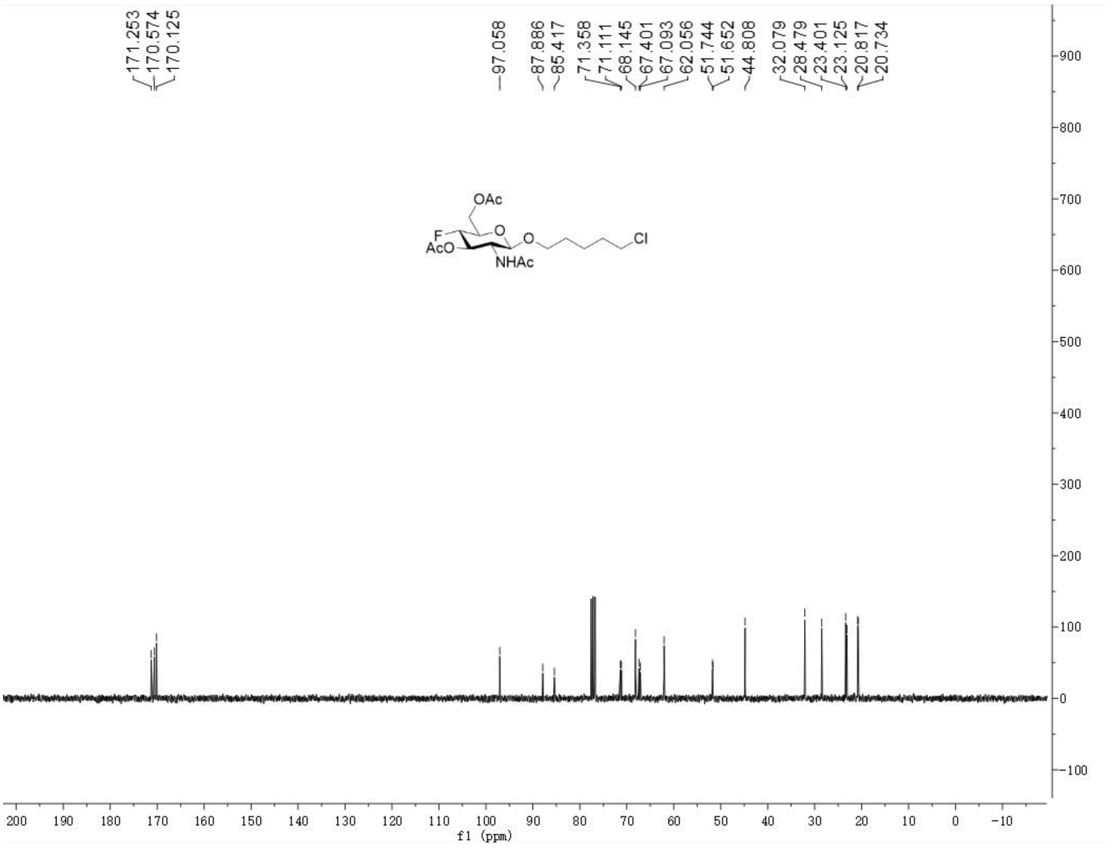

^{13}C NMR spectrum of compound **13**.

^{1}H NMR and ^{13}C NMR for compound 14.

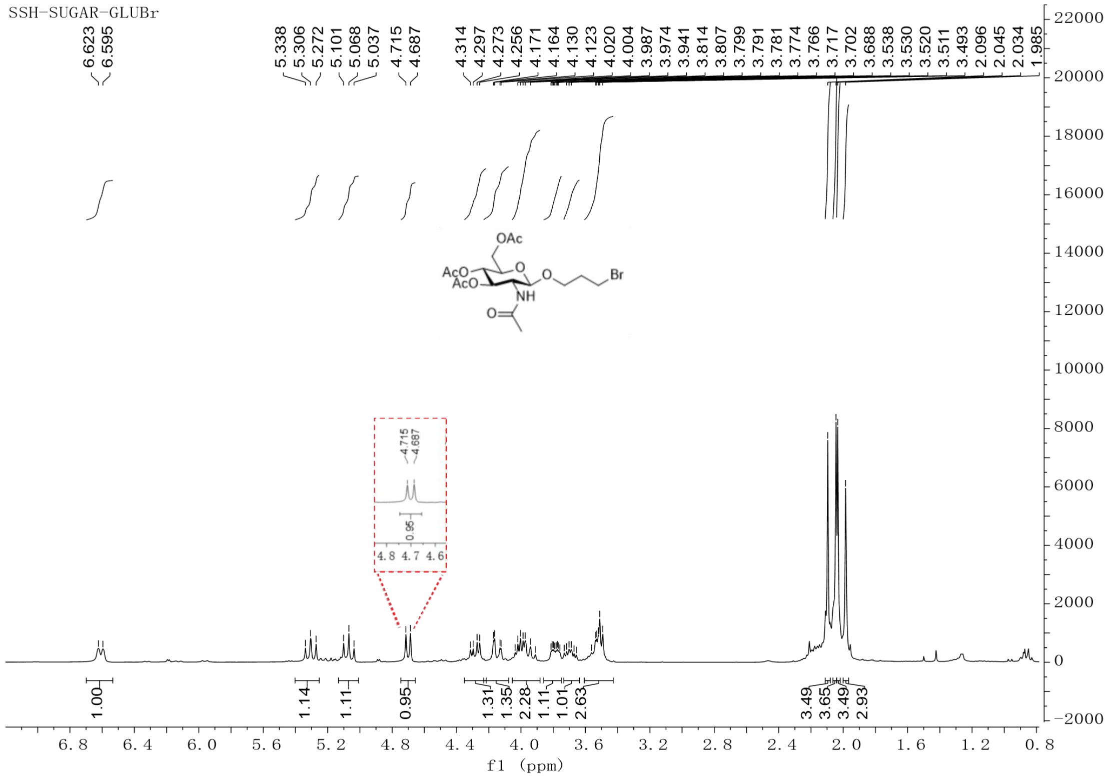

^{1}H NMR spectrum of compound **14**.

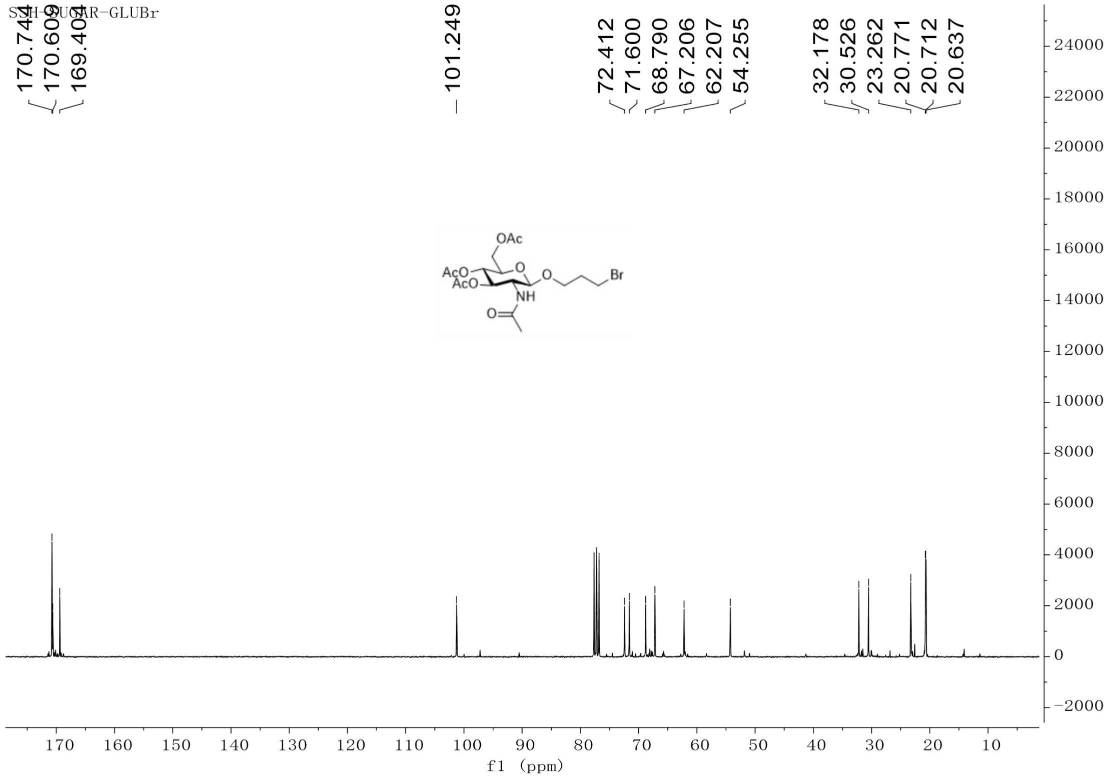

^{13}C NMR spectrum of compound **14**.

^{1}H NMR and ^{13}C NMR for compound 15.

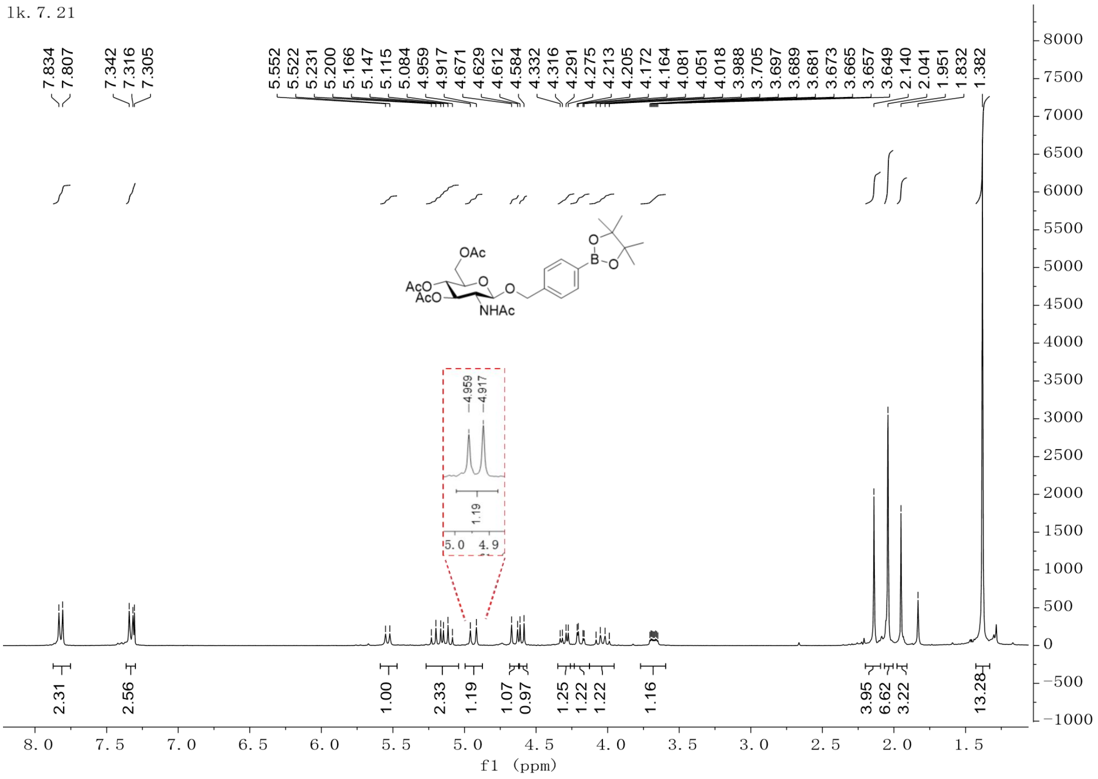

^{1}H NMR spectrum of compound **15**.

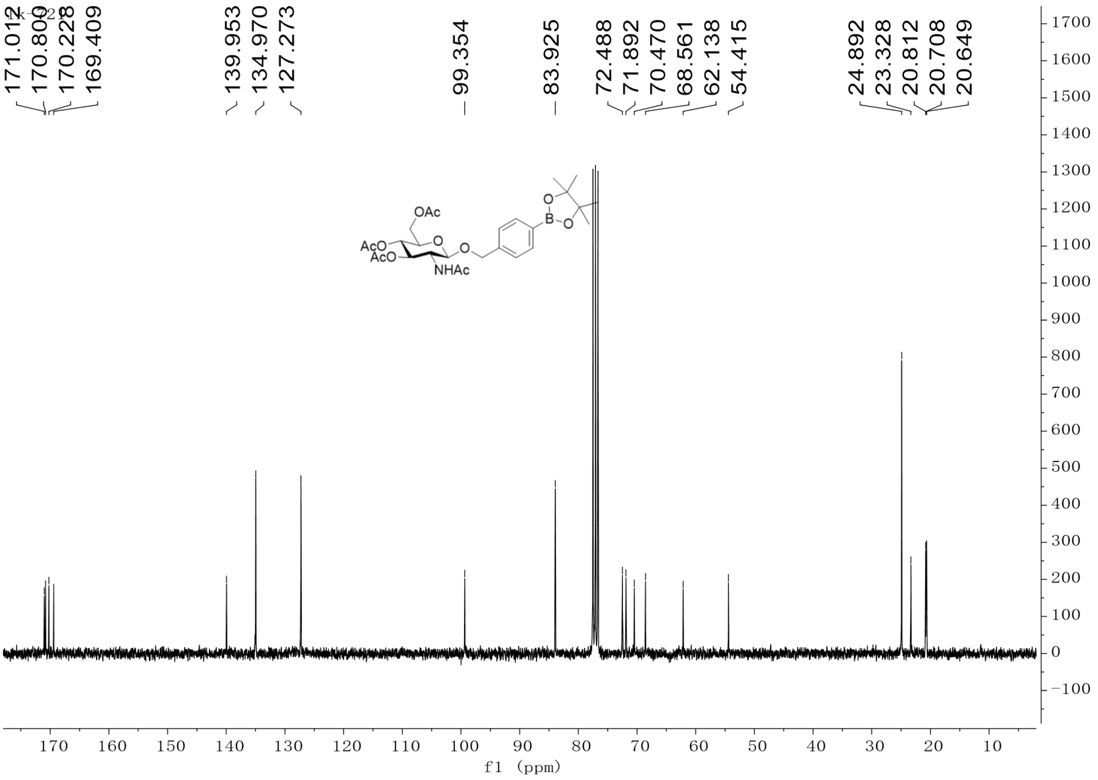

^{13}C NMR spectrum of compound **15**.

^{1}H NMR and ^{13}C NMR for compound 18.

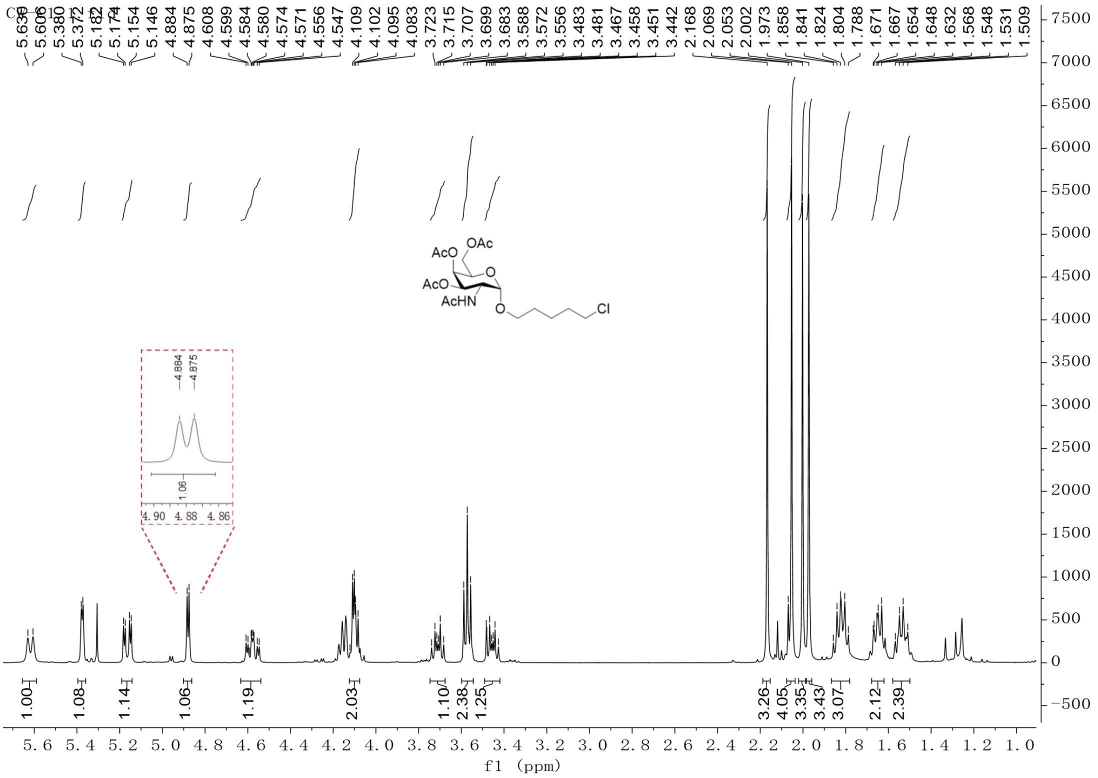

^{1}H NMR spectrum of compound **18**.

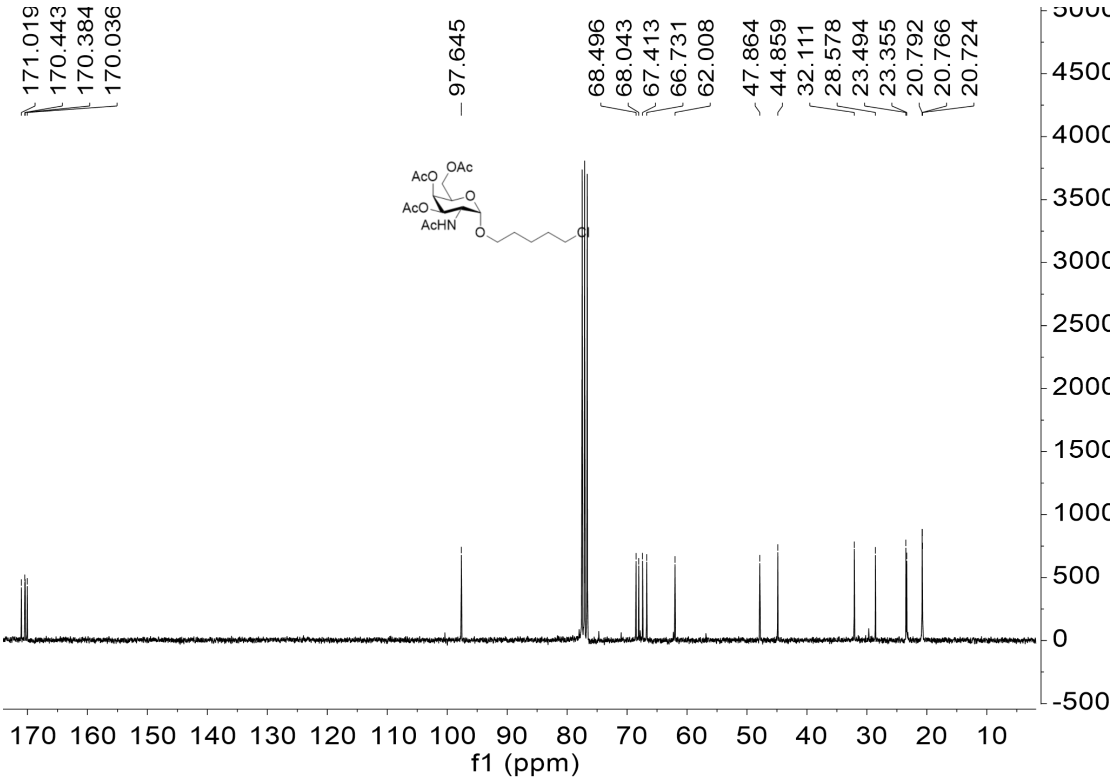

^{13}C NMR spectrum of compound **18**.

^{1}H NMR and ^{13}C NMR for compound 19.

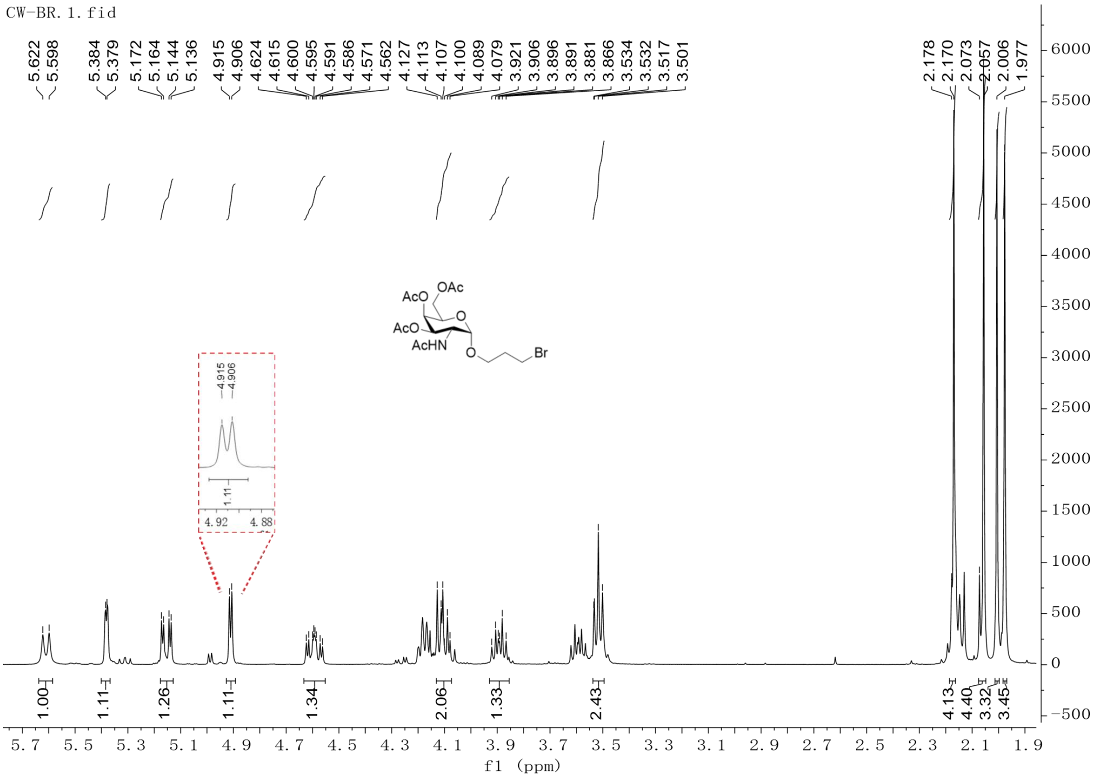

^{1}H NMR spectrum of compound **19**.

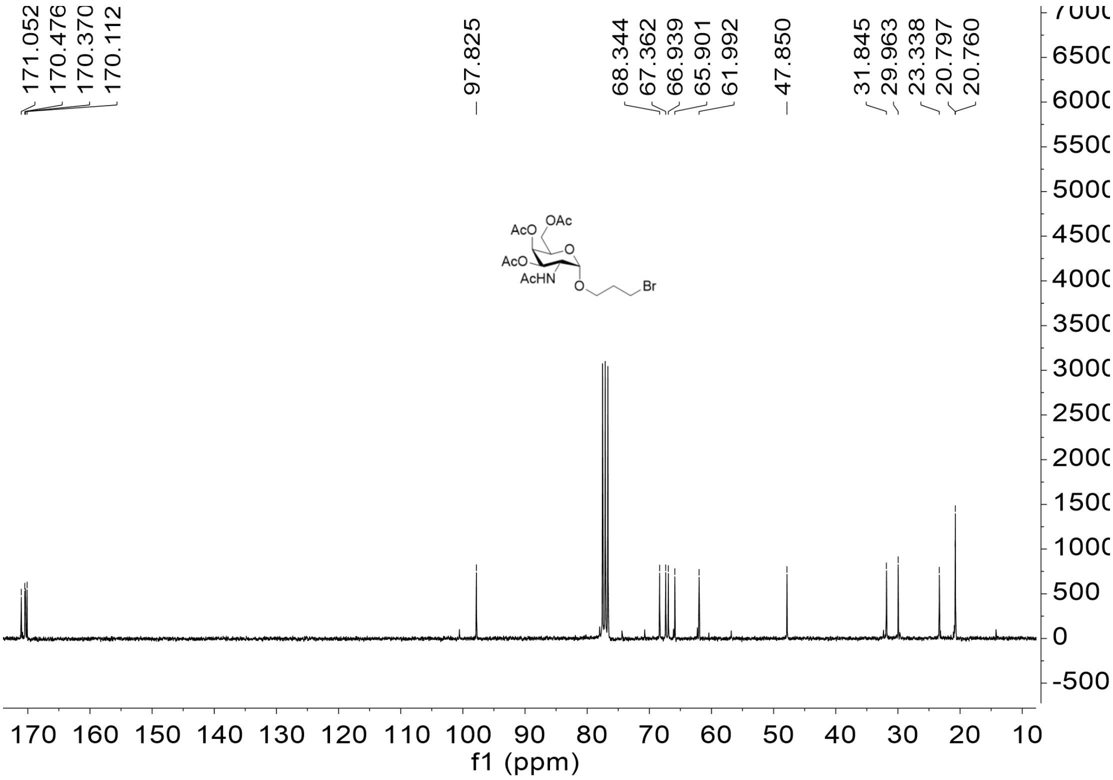

^{13}C NMR spectrum of compound **19**.

^{1}H NMR and ^{13}C NMR for compound 20.

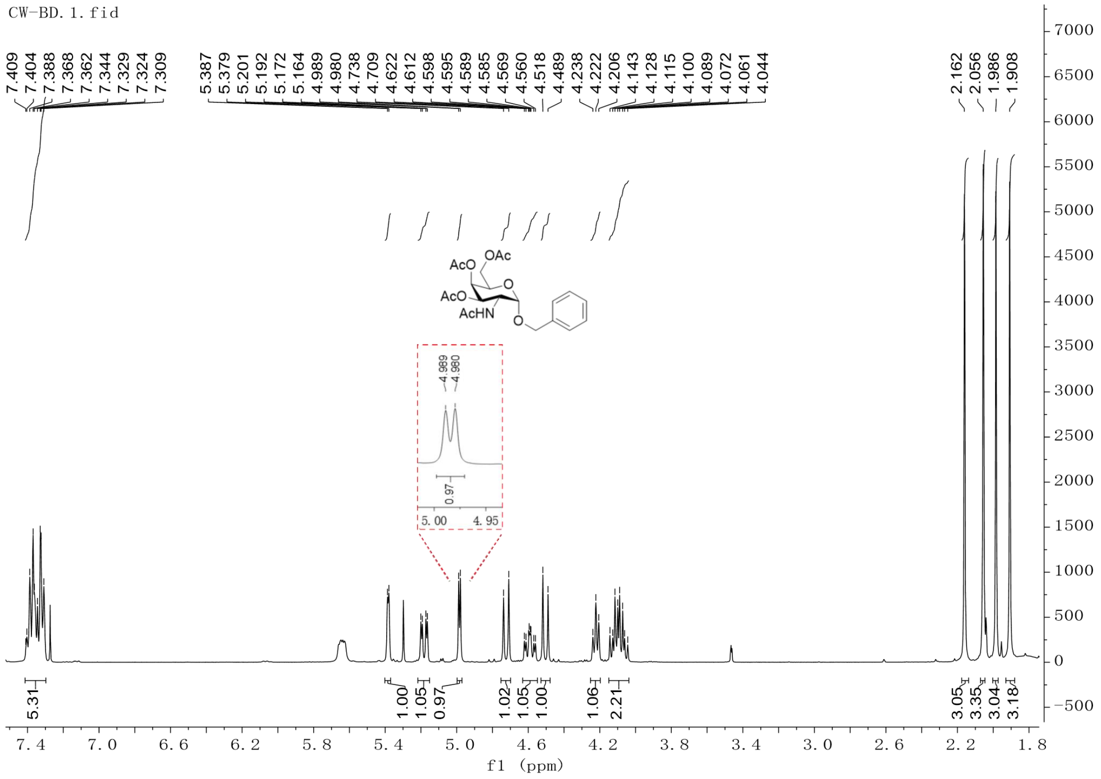

^{1}H NMR spectrum of compound **20**.

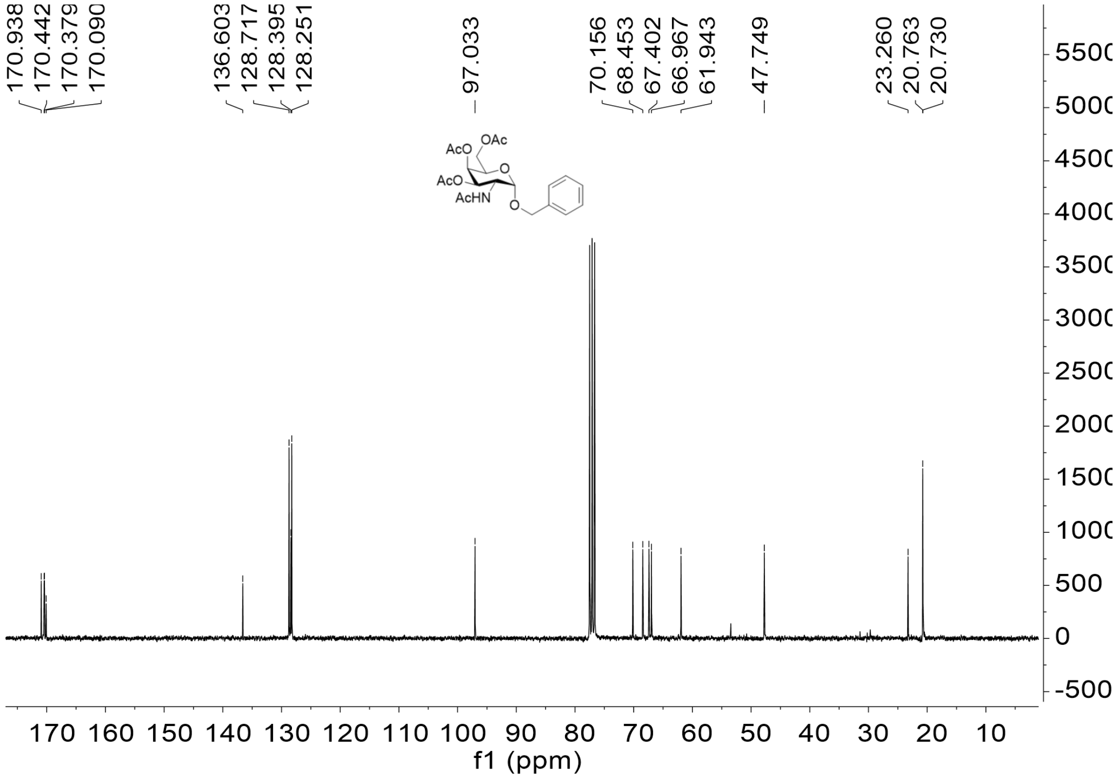

^{13}C NMR spectrum of compound **20**.

^{1}H NMR and ^{13}C NMR for compound 21.

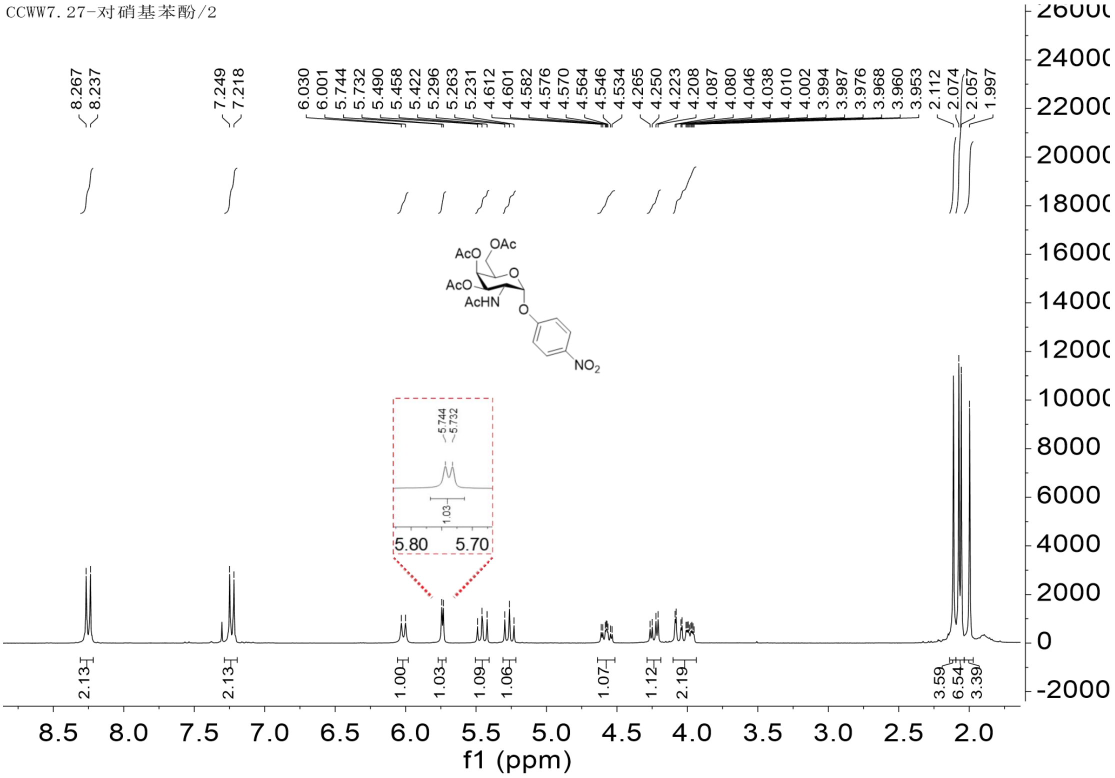

^{1}H NMR spectrum of compound **21**.

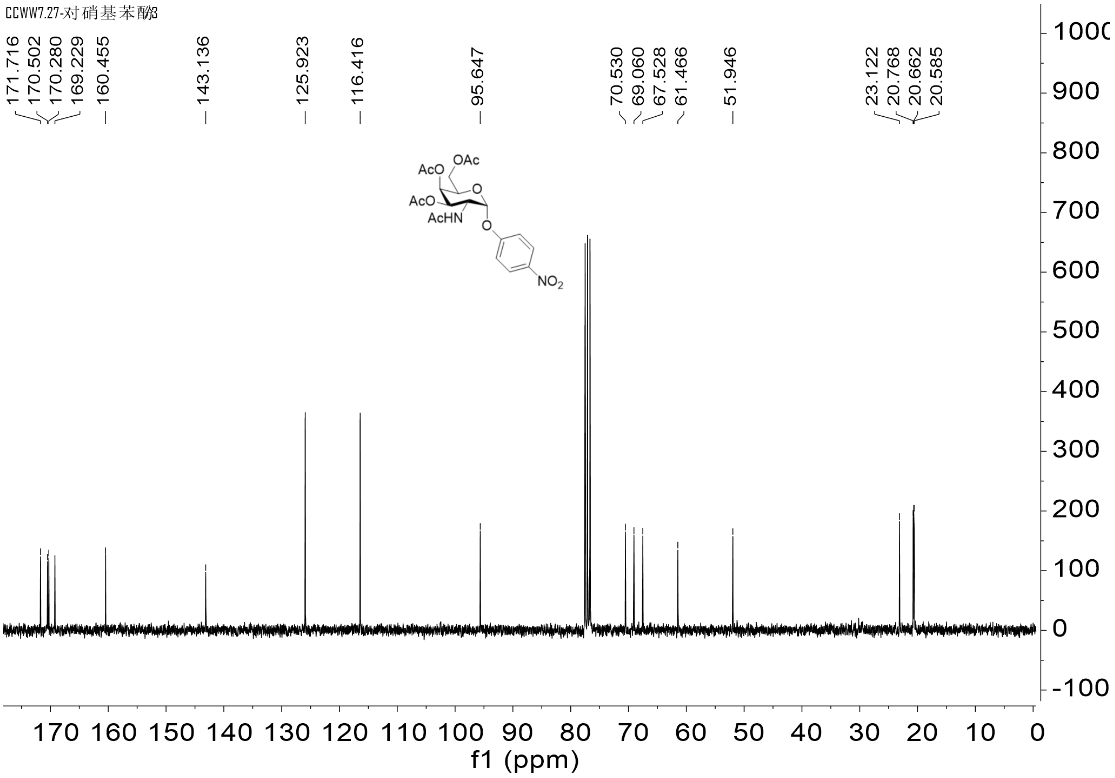

^{13}C NMR spectrum of compound **21.**

^{1}H NMR and ^{13}C NMR for compound 22.

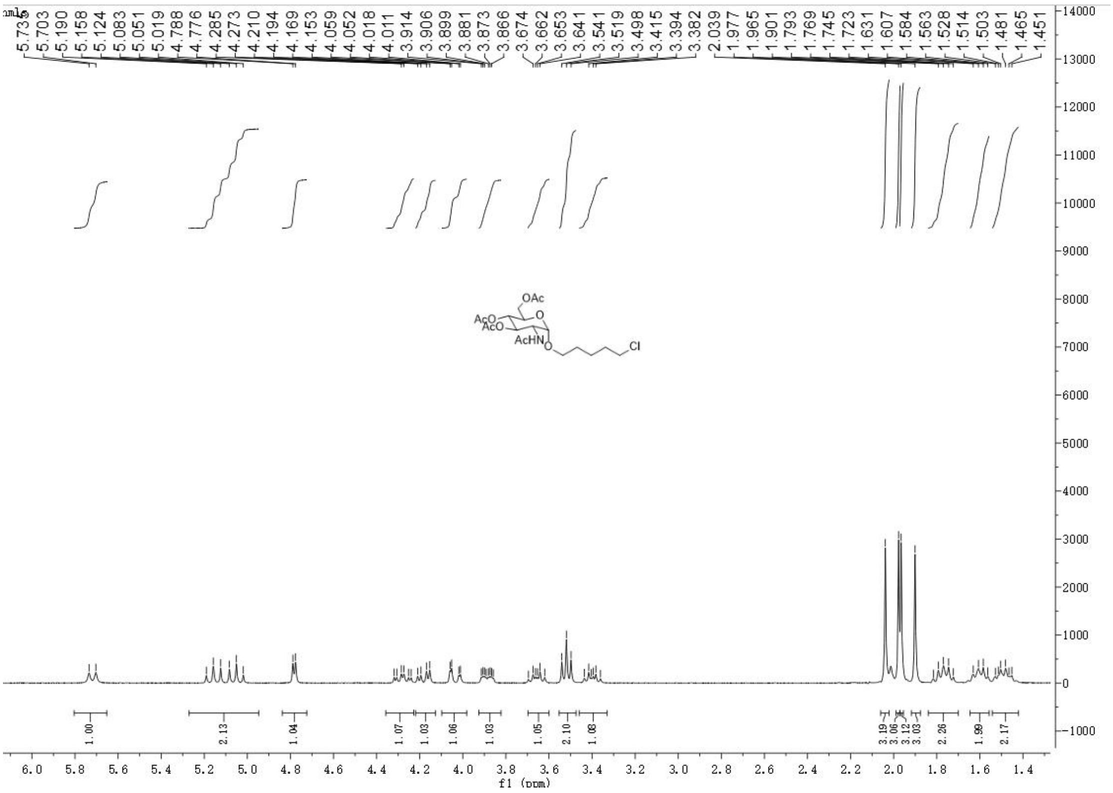

^{1}H NMR spectrum of compound **22**.

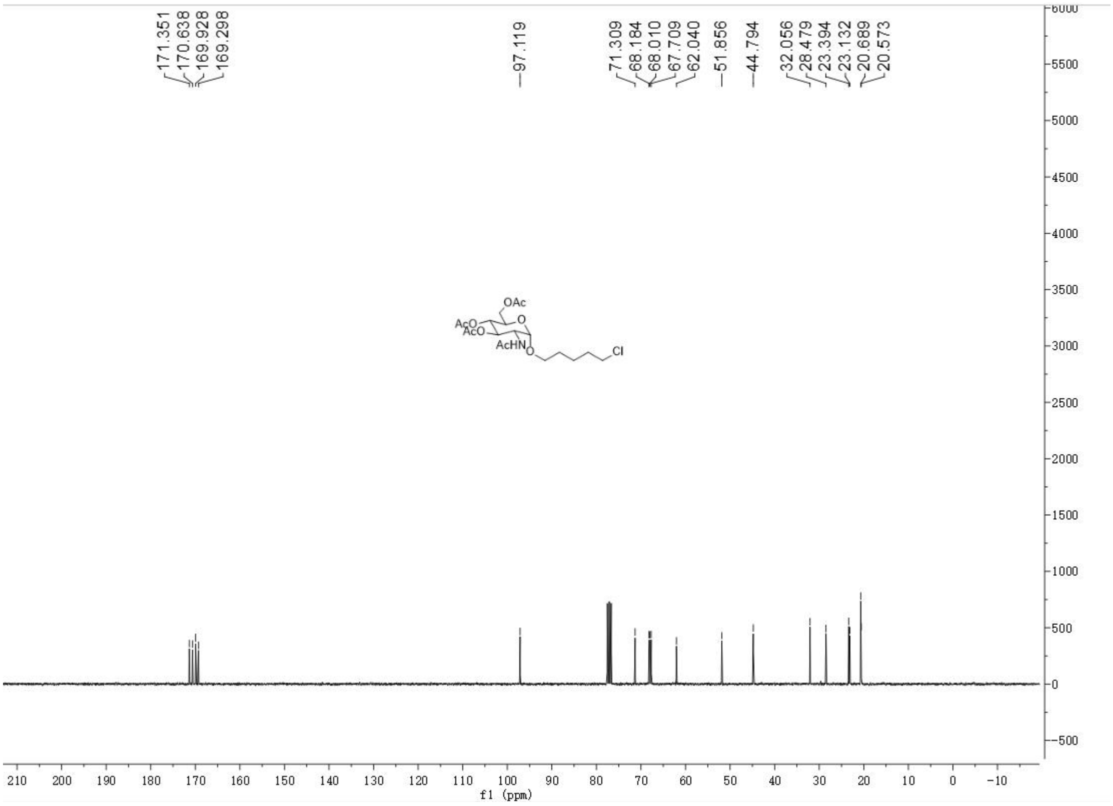

^{13}C NMR spectrum of compound **22.**

^{1}H NMR and ^{13}C NMR for compound 27.

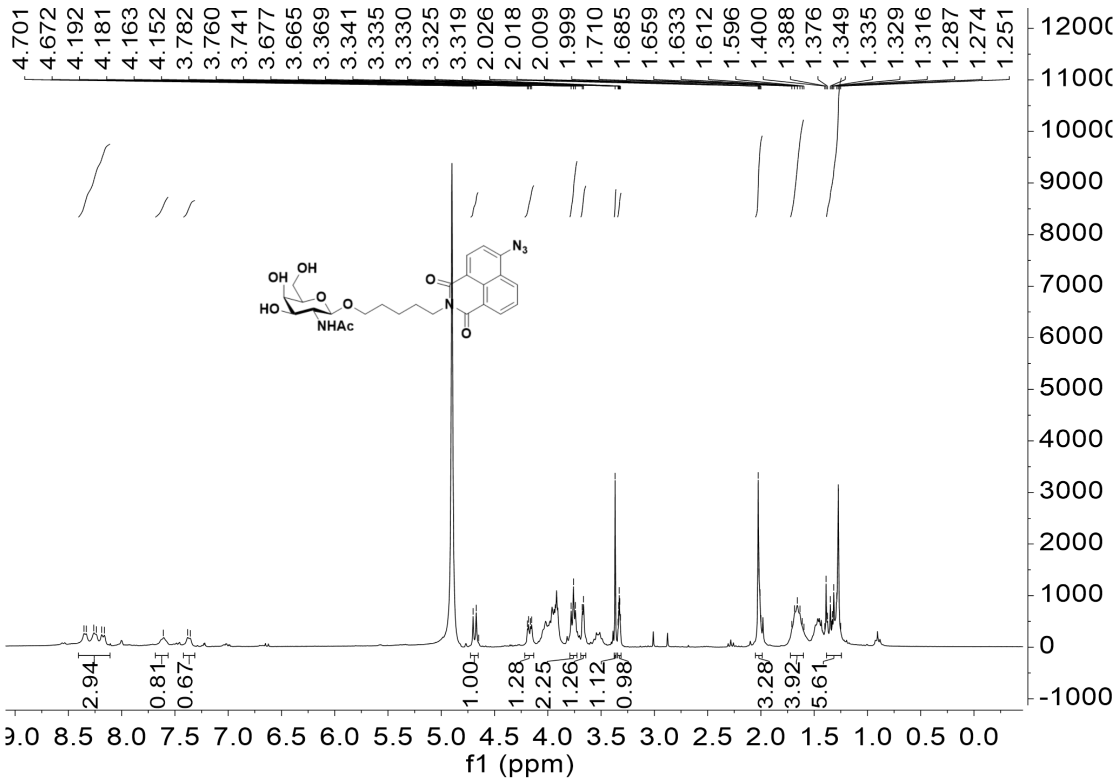

^{1}H NMR spectrum of compound **27**.

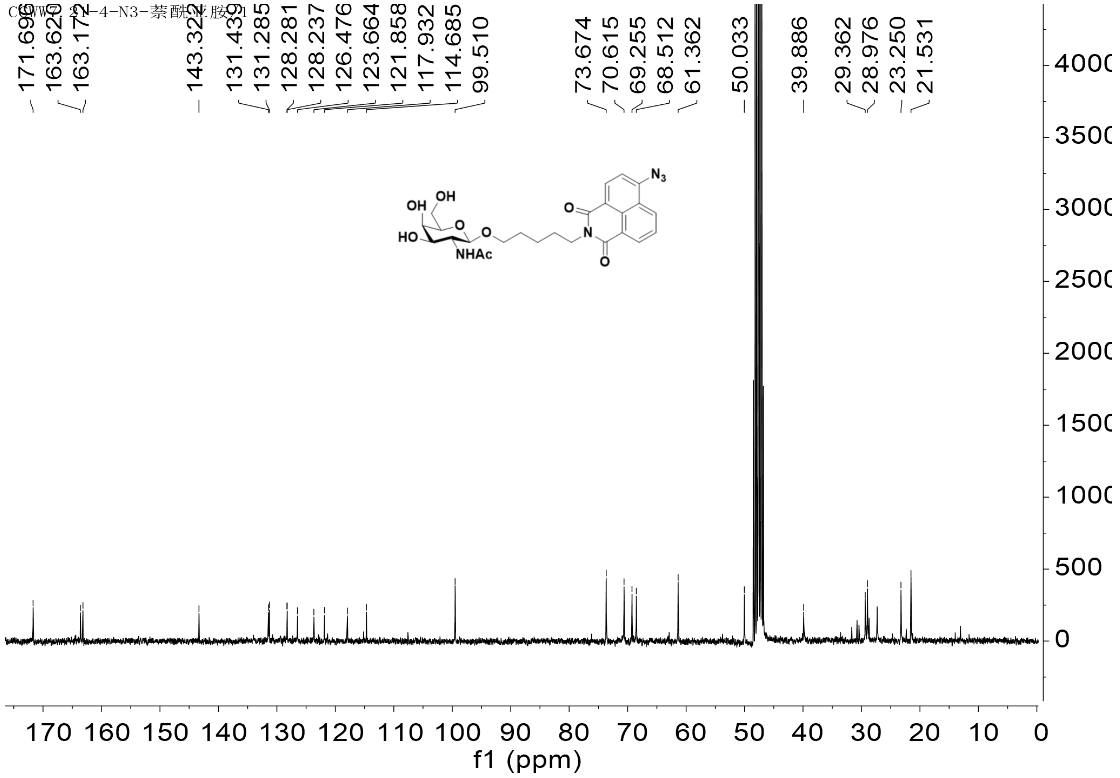

^{13}C NMR spectrum of compound **27.**
